# A blood-based DNA damage signature in patients with Parkinson’s disease is associated with disease progression

**DOI:** 10.1038/s43587-025-00926-x

**Published:** 2025-09-05

**Authors:** Daisy Sproviero, César Payán-Gómez, Chiara Milanese, Sander Barnhoorn, Shixiang Sun, Akos Gyenis, Domenico Delia, Tammaryn Lashley, Jan H. J. Hoeijmakers, Jan Vijg, Pier G. Mastroberardino

**Affiliations:** 1 IFOM-ETS, The AIRC Institute of Molecular Oncology, Milan, Italy; 2 Universidad Nacional de Colombia, Sede de La Paz, La Paz, Colombia; 3 Departmental Faculty of Medicine and Surgery, UniCamillus-Saint Camillus International University of Health and Medical Sciences, Rome, Italy; 4 Department of Molecular Genetics, Erasmus MC, Rotterdam, the Netherlands; 5 Department of Genetics, Albert Einstein College of Medicine, Bronx, NY USA; 6 Institute for Genome Stability in Ageing and Disease, Cluster of Excellence for Aging Research, Faculty of Medicine, University of Cologne, Cologne, Germany; 7 The Queen Square Brain Bank for Neurological Disorders, Department of Clinical and Movement Neuroscience, UCL Queen Square Institute of Neurology, London, UK; 8 Department of Neurodegenerative Diseases, UCL Queen Square Institute of Neurology, London, UK; 9 Princess Maxima Center for Pediatric Oncology, Oncode Institute, Utrecht, the Netherlands; 10 Center for Single-Cell Omics, School of Public Health, Shanghai Jiao Tong University School of Medicine, Shanghai, China; 11 Universita’ degli Studi dell’Aquila, L’Aquila, Italy

**Keywords:** Parkinson's disease, Predictive markers, Ageing

## Abstract

Aging is the main risk factor for Parkinson’s disease (PD), yet our understanding of how age-related mechanisms contribute to PD pathophysiology remains limited. We conducted a longitudinal analysis of blood samples from the Parkinson’s Progression Markers Initiative cohort to investigate DNA damage in PD. Patients with PD exhibited disrupted DNA repair pathways and biased suppression of longer transcripts, indicating age-related, transcription-stalling DNA damage. Notably, at the intake visit, this DNA damage signature was detected only in patients with more severe progression of motor symptoms over 3 years, suggesting its potential as a predictor of disease severity. We validated this signature in independent PD cohorts and confirmed increased DNA damage in peripheral blood cells and dopamine neurons of the substantia nigra pars compacta in postmortem PD brains. Our study sheds light on an aging-related mechanism in PD pathogenesis and identifies potential markers of disease progression, providing a diagnostic platform to prognosticate disease progression.

## Abstract (web summary)

Aging is a risk factor for Parkinson’s disease; however, how DNA damage accumulation, a hallmark of aging, contributes to its pathophysiology remains incompletely understood. Here, the authors identify a blood-based DNA damage signature that is associated with disease progression in patients with Parkinson’s disease.

## Main

Parkinson’s disease (PD) is a common neurodegenerative disorder that principally, but not exclusively, affects dopaminergic neurons in the nigrostriatal circuits. PD is primarily idiopathic (iPD), with less than 10% of cases attributed to monogenic mutations. However, the genetic forms have provided insight into the intricate pathogenic mechanisms underlying disease progression, which include perturbations in protein homeostasis, oxidoreductive balance, mitochondrial function and intracellular trafficking^1,2^. These alterations often interact in a complex manner, resulting in the characteristic motor symptoms observed in PD. The disease, however, is remarkably heterogeneous^3^. Patients display significant variability in both clinical presentation and progression, which is attributed to differences in the underlying pathophysiological processes^4^. Consequently, PD has also been considered a syndrome with several disease subtypes^5,6^. Nonetheless, our understanding of the causes of PD heterogeneity is extremely rudimentary, and the stratification of patients into homogeneous groups based on the expected clinical presentation and disease progression remains unachievable at present. This drawback represents a major confounder in clinical trials, as treating a heterogeneous cohort of patients is likely to yield varied outcomes^7,8^. Conversely, stratifying patients into clinically homogeneous groups would facilitate the development of personalized and more effective treatments.

The principal risk factor for PD is aging^9^. However, how the biology of aging contributes to PD pathophysiology is poorly understood. A fundamental and causative mechanism of aging is the progressive accumulation of damage in nuclear DNA^10^. Nuclear DNA is constantly exposed to exogenous and endogenous factors that induce chemical modifications of its bases, ultimately compromising the fidelity of genetic information. These chemical alterations affect cellular function (for instance, causing quantitative and qualitative perturbations in transcription) and culminate in transmissible mutations in replicating cells. The impact of DNA damage on transcription is highly relevant for nondividing, postmitotic neurons. Given the severe consequences of DNA damage accumulation, evolution has equipped all organisms with a highly efficient and intricate network that promptly corrects these lesions^11^.

Nuclear DNA damage can explain most, if not all, hallmarks of aging and has been considered the ultimate underlying cause of age-related systemic functional loss and diseases^12^. Severe defects in DNA repair systems cause progeroid diseases, which are characterized by the premature appearance of multiple symptoms of aging, in humans and mice. Meanwhile, natural aging is associated with an increased burden of somatic mutations in virtually all organs and tissues, including the brain and neurons in particular^13,14^, which also points toward increased nuclear DNA damage as its underlying cause.

Impaired DNA repair has also been hypothesized to contribute to the pathogenesis of age-related neurodegenerative diseases^15^, and multiple pieces of evidence point to a role in PD. Peripheral fibroblasts from patients with PD display reduced DNA repair capacity^16^, mouse models with mild defects in DNA repair exhibit impairment in the nigrostriatal dopaminergic pathways^16^, and synucleinopathy is associated with the activation of the DNA damage response in mice^17^. Moreover, meta-analysis data available in the PDGene database (https://www.pdgene.org/)^18,19^ indicate that the nucleotide excision repair (NER) gene *ERCC8* may be associated with PD (*P* = 9.27 × 10^−07^, odds ratio 1.15, 95% confidence interval 1.08–1.21 for the rs11744756 polymorphism). Finally, a role for DNA damage accumulation in PD is also supported by evidence demonstrating that certain patients with genetic diseases caused by defects in DNA repair manifest levodopa-responsive dopaminergic symptoms^20,21^. Collectively, these findings suggest that the accumulation of nuclear DNA damage may have a role in PD pathogenesis. However, evidence to prove this hypothesis is inconclusive thus far.

In this study, we leveraged the large dataset collection from the Parkinson’s Progression Markers Initiative (PPMI) to gather additional bioinformatic evidence demonstrating a role for nuclear DNA damage in PD.

## Results

### Patients with PD show dysregulation in pathways related to RNA processing, transcription and translation

**Fig. 1:**
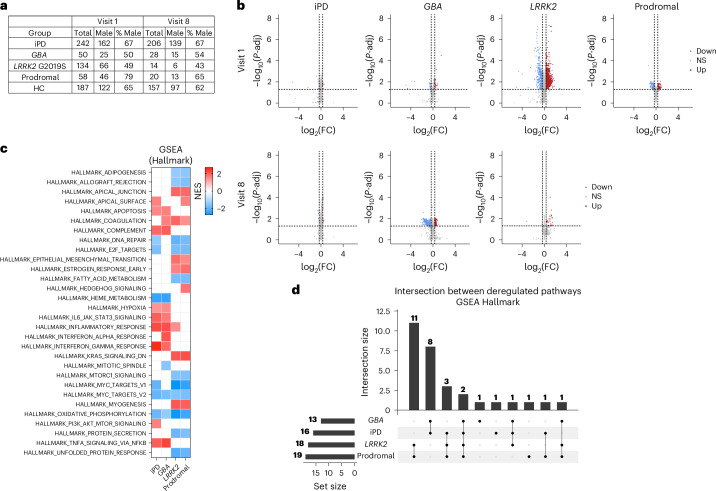
Common deregulated pathways among iPD, *GBA*, *LRRK2* and prodromal cases from blood transcriptomic data at visit 1. **a**, Demographic table summarizing the number and sex of patients in the four PD subgroups and individuals in the HC group at both time points. **b**, Volcano plots of DEGs in patients versus controls in the PPMI cohort. Genes with *P*-adj < 0.05 and log_2_(FC) > 0.322 are shown in red; genes with log_2_(FC) < −0.322 are shown in blue. The criteria log_2_(FC) > |0.322| and *P*-adj < 0.05, as calculated with the Wald test using DESeq2, were consistently applied throughout the study. Down, downregulated; Up, upregulated; NS, not significant. **c**, GSEA showing pathway alterations against the Hallmark collection in iPD, *GBA* and *LRRK2* G2019S carriers, and prodromal cases at visit 1. Upregulated and downregulated processes are marked in red and blue, respectively. White boxes indicate that the process is not altered. Upregulated processes were largely related to inflammatory processes, whereas downregulated processes predominantly concerned macromolecular synthesis linked to transcription and translation. Significance was set at *P*-adj < 0.05. **d**, UpSet plot of enriched GSEA Hallmark pathway intersections among datasets, revealing that two pathways (HALLMARK_OXIDATIVE_PHOSPHORYLATION and HALLMARK_MYC_TARGETS V2) were shared among the studied groups at visit 1. In the heatmaps, upregulated and downregulated pathways are represented in red and blue, respectively; *P*-adj < 0.05. In GSEA, enrichment significance was assessed using a one-sided, permutation-based test on the NES, as implemented in the fgseaMultilevel function, which uses an adaptive multilevel Monte Carlo algorithm and includes multiple comparison corrections to estimate low *P* values efficiently.

Taking advantage of the PPMI resource (https://www.ppmi-info.org), we evaluated longitudinal expression data in blood samples from 484 patients with PD and 187 healthy control (HC) participants examined at the intake visit (visit 1) and 268 patients with PD and 157 HCs examined in a follow-up visit after 36 months (visit 8) (Fig. 1a; detailed information in Supplementary Tables 1 and 2; see also Supplementary Table 10 for an index of the tables’ contents). This cohort consisted of patients with iPD, patients carrying genetic mutations in *LRRK2* and *GBA*, HC individuals and prodromal cases. As determined by the PPMI inclusion criteria, the prodromal group consisted of male and female individuals older than 60 years who had no motor symptoms but had a higher risk of developing PD due to the presence of one or more of the following signs: hyposmia at or below the 10th percentile as determined by the University of Pennsylvania Smell Identification Test, polysomnography results consistent with or a clinical diagnosis of rapid eye movement behavior disorder, and mild dopaminergic deficits as evidenced by DaTscan (visualization of dopamine transporter levels using single-photon emission computed tomography) or VMAT-2 imaging (visualization of vesicular monoamine transporter type 2 levels using positron emission tomography). Analyses were performed on 53 of the 58 prodromal individuals who were clinically stable and did not develop PD symptoms within 2 years, as indicated in the PPMI database. No information was available for the remaining five cases, which were therefore excluded from the study.

**Extended Data Fig. 1:**
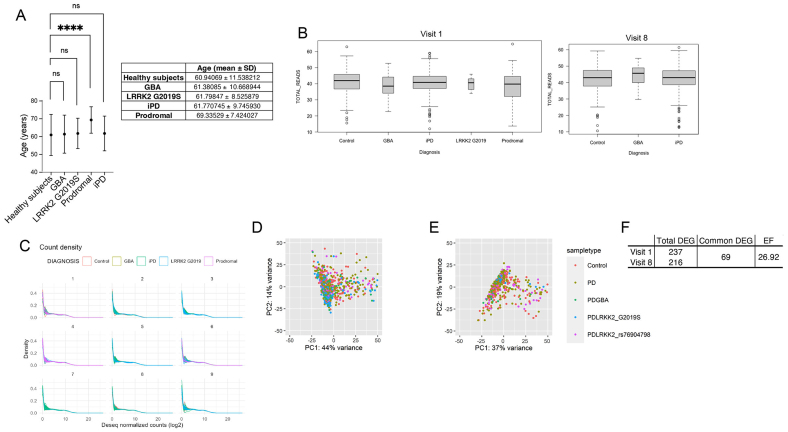
Cohorts and quality control. **(a)** Demographic tables summarizing number and sex of patients in the four PD subgroups and healthy controls at both timepoints (Plots show mean ± SD). Aging of PD patients’ groups is comparable with that of healthy subjects, as indicated in the graph (left) and in the table (right). Prodromal patients, however, are significantly older than healthy subjects (**** P < 0.0001, Kruskal Wallis H-test). (**b**) Total read counts of healthy controls, GBA, iPD, *LRRK2*; and prodromals are comparable (Kruskal-Wallis chi-squared = 4.8184, df = 4, p-value = 0.3064 for Visit 1; Kruskal-Wallis chi-squared = 10.442, df = 4, p-value = 0.0336 for Visit 8). (**c)** Density plot of the DESeq normalized gene counts for all the samples used. (**d, e**) Principal component analysis plots. (**f**) Table illustrating the enrichment factor of common deregulated genes at visit 1 and visit 8.

Age was comparable between patients with PD and HCs, as indicated by the Kruskal–Wallis test; therefore, the observed effects are genuinely attributable to disease effects rather than age differences. However, as prodromal cases were significantly older than the controls, we performed correction using age as a covariate in all analyses involving this group (Extended Data Fig. 1a).

After conducting initial quality control analyses to identify potential biases or low-quality samples, we analyzed datasets from patients examined at visit 1 and after 36 months (visit 8). Quality control revealed a homogeneous distribution of gene counts (Extended Data Fig. 1b,c) and comparable library sizes. Gene counts were obtained using Salmon, a tool designed for fast and accurate quantification of transcript expression from RNA-sequencing data. Salmon operates through a quasi-mapping approach that assigns RNA-sequencing reads to transcripts without performing full alignments. It uses a two-phase inference procedure that corrects for various biases, such as transcript length and sequence composition. Therefore, the method ensures precise and reliable quantification of gene expression^22^.

Principal component (PC) analysis (PCA) revealed a compact clustering of data in all experimental groups. In patients with genetic PD and those with iPD, 44% of the variance was attributable to PC1 at baseline; at visit 8, PC1 accounted for 37% of the variance (Extended Data Fig. 1d,e). The compact clustering observed in the PCA plots among the experimental groups—that is, iPD, *LRRK2* and *GBA* mutants, and HCs—aligns with previous reports and supports the reliability of our expression analysis^23^.

**Extended Data Fig. 2:**
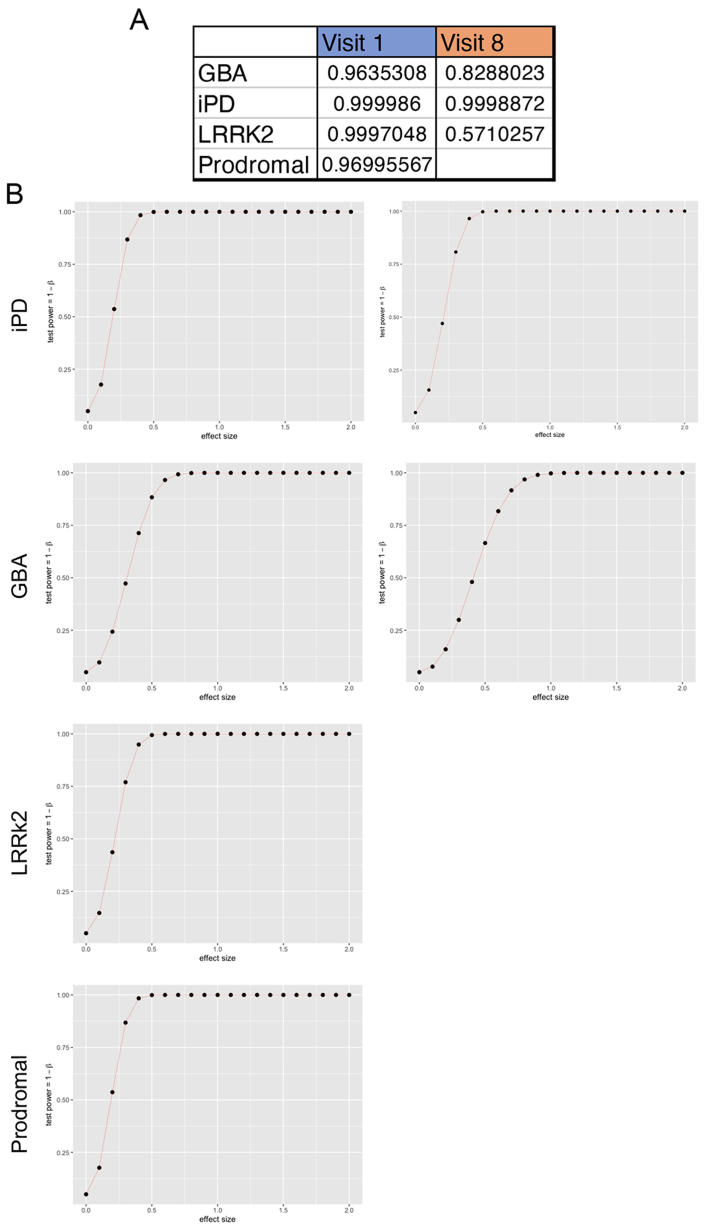
Retrospective post-hoc Power Analysis. (**a**) Power of the different experimental groups when compared to the healthy subjects control group with a medium-large effect size (0.6). (**b**) Power-versus-effect size curves of the different experimental groups at visit 1 and visit 8.

To assess data quality further, we compared the transcriptome of patients with PD at visits 1 and 8 against that of HCs and calculated the enrichment factor for common deregulated transcripts (Supplementary Table 3). There were 69 common differentially expressed genes (DEGs) between the two visits, and enrichment factor analysis revealed that this overlap was 26-fold higher than what was expected by chance, substantiating the reliability of the datasets (Extended Data Fig. 1f). We performed a retrospective post hoc power analysis and confirmed that the experimental groups were sufficiently powered to reveal differences in the transcriptomes of all patients with PD at both time points, except for the *LRRK2* G2019S group at visit 8, which was therefore excluded from further analyses (Extended Data Fig. 2a,b).

**Extended Data Fig. 3:**
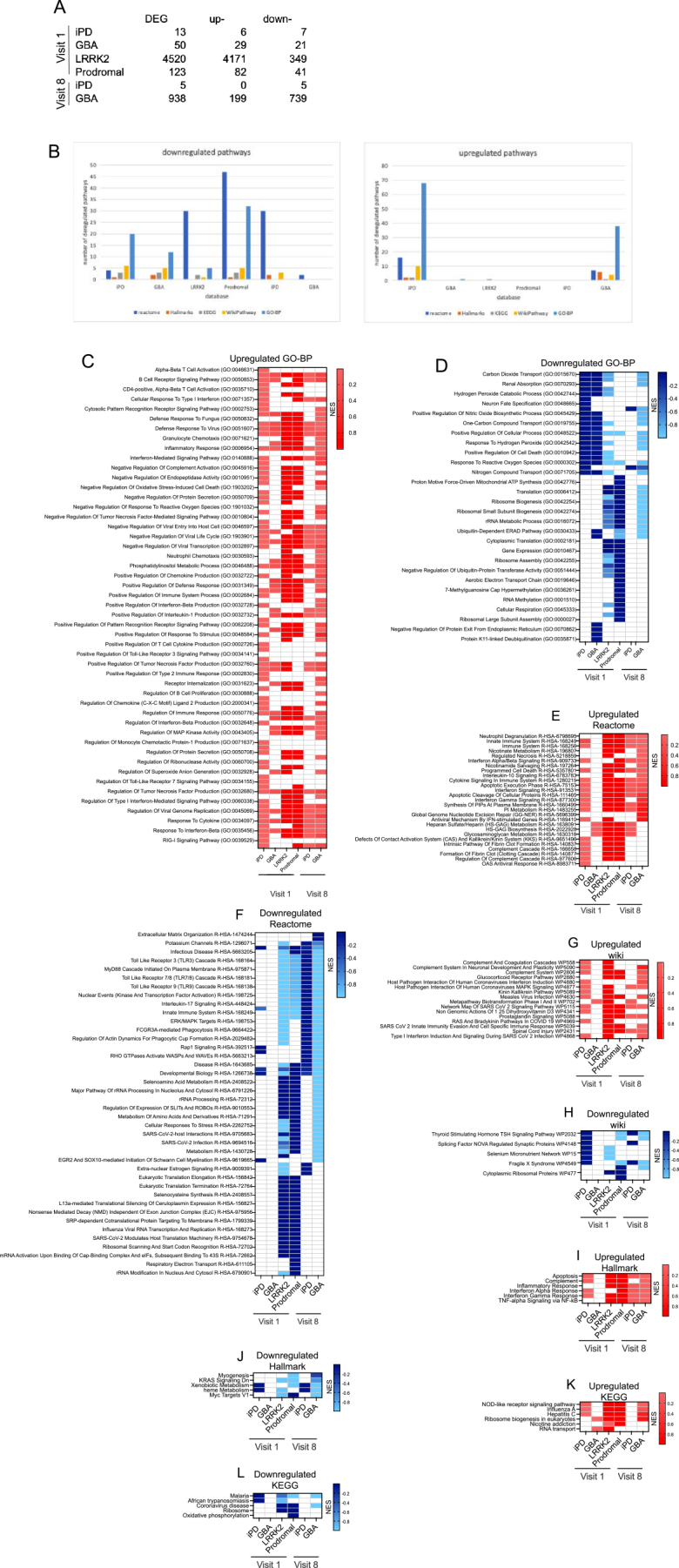
Over-representation analysis of significantly DEG in PD patients’ blood. **(a**) Table summarizing the number of significantly total, up-, and down-regulated DEG in the analyzed experimental groups, at visit 1 and visit 8 ((log_2_FC>|0.322|, that is 1.25 FC, and adj.p-value < 0.05). (**b**) Bar graph illustrating of the number of deregulated pathways detected performing ORA against different gene collections. (**c, d**) ORA based on the GO-BP collection for sufficiently powered groups at visit 1 (iPD, GBA and *LRRK2* G2019S carriers, and prodromal cases’) and at visit 8 (iPD and GBA carriers) at visit 8. (**e**-**m**) ORA of significantly deregulated genes using the additional collection Reactome (**e**, **f**), Wikipathways (**g**, **h**), Hallmark(**i**, **j**), and KEGG (**k**, **l**). Upregulated pathways are represented in red, downregulated pathways in green.

Volcano plot representation confirmed the findings of previous studies characterizing the PPMI cohort^23^, showing that DEGs exhibited modest fold changes (FCs), although their statistical significance was highly pronounced at both visits, as indicated by the small adjusted *P* (*P*-adj) values (Fig. 1b and Supplementary Table 3). The DEG number (log_2_(FC) > |0.322| (that is, an FC of 1.25) and *P*-adj < 0.05 were consistently applied throughout the study) significantly varied between experimental groups and time points, indicating heterogeneity among different forms of PD (Fig. 1b, Extended Data Fig. 3a and Supplementary Table 3).

To gain initial insights into the molecular processes altered in PD and its different forms, we performed overrepresentation analysis (ORA) based on significant DEGs^24^. At present, several pathway repositories are available, and there is evidence indicating that reliance on a single collection may bias the analysis^25^. Therefore, we analyzed the data against five different collections: Reactome, the Molecular Signatures Database (MSigDB) Hallmark Gene Set Collection, the Kyoto Encyclopedia of Genes and Genomes (KEGG), WikiPathways^26–29^ and the Gene Ontology (GO) knowledgebase to infer altered biological processes (GO-BP)^30,31^.

ORA confirmed significant heterogeneity among the experimental groups, revealing variability in the number of deregulated pathways (Extended Data Fig. 3b–l). The analysis also confirmed findings reported in previous studies; for instance, our results showed that upregulated pathways were largely related to inflammation (Extended Data Fig. 3e,g,i,k and Supplementary Tables 3 and 4). However, several experimental groups did not show any alterations (Extended Data Fig. 3b). In general, the representation of DEGs in the gene set composing the identified pathways (that is, the overlap) was small (Supplementary Table 3). These observations are consistent with the small magnitude changes reported in previous studies based on the PPMI cohort^23^ and have also been described in other chronic human diseases, where the subtle contributions of a large number of genes dictate the phenotype^32^. In fact, ORA based on the *P*-adj values of genes is more suited to detect larger differences and is therefore suboptimal for analyzing the PPMI datasets, as indicated by the volcano plots in Fig. 1b and also suggested by previous studies that, differently from our log_2_(FC) > |0.322| cutoff, applied a threshold of log_2_(FC) > |0.1| (refs. ^23,33^).

ORA’s lack of sensitivity in detecting small effects is a significant weakness because functional differences could stem from small changes in groups of related genes that are coordinately expressed (that is, expression modules), which cannot be detected by a method limited to significant DEGs. This drawback is addressed by gene set enrichment analysis (GSEA)^34^, which uses all the ranked genes in the datasets according to their individual statistical significance to calculate the aggregated statistical significance of a set of related genes (that is, a pathway).

We initially focused on early PD cases (that is, from visit 1) and interrogated the Hallmark database because it reduces both the redundancy and heterogeneity intrinsic to large gene set compendia. The Hallmark collection offers a comprehensive yet synthetic depiction of the molecular landscape. GSEA detected deregulation in expected pathways, for instance, upregulation of inflammatory processes and downregulation of oxidative phosphorylation (Fig. 1c and Supplementary Table 5). The analysis also confirmed heterogeneity among the patient groups (iPD, *GBA*, *LRRK2*) and prodromal individuals, unraveling differences in the molecular landscape; in fact, the four experimental groups shared only two pathways, HALLMARK_MYC_TARGETS_V2 and HALLMARK_OXIDATIVE_PHOSPHORYLATION, both of which were downregulated (Fig. 1c,d).

To examine at a more granular level these two common deregulated pathways at visit 1, we took advantage of leading-edge gene (LEG) analysis. GSEA identifies the subset of genes (that is, LEGs) that, for each experimental group, provide the highest contribution to the enrichment signal of a given pathway^34^. Therefore, LEG analysis reduces the dimensionality of enriched gene sets (that is, several of the GSEA-identified pathways may contain the same genes), providing a focused depiction of transcriptomic changes and facilitating the identification of crucial biological processes.

**Extended Data Fig. 4:**
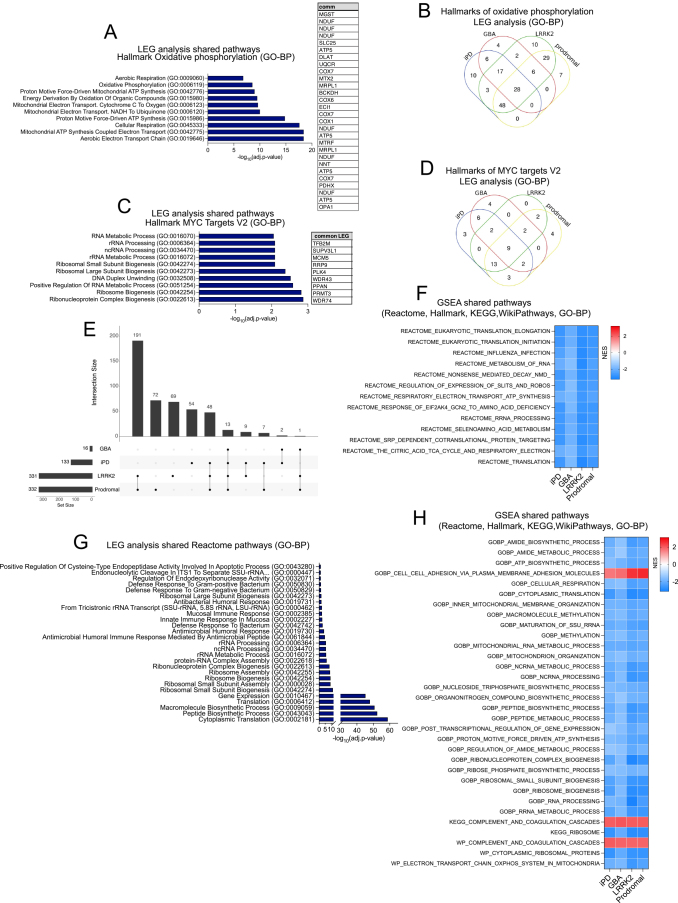
Leading-edge gene (LEG) analysis of iPD, GBA, *LRRK2* G2019S, and prodromal cases at visit1. (**a**) Bar plot of LEG analysis dissecting the key contributors in the shared HALLMARK_OXIDATIVE_PHOSPHORYLATION. Genes in this pathway, which are altered in all the four experimental groups, relate to mitochondrial respiration. (**b**) Venn diagrams illustrating the relationship of the genes in the HALLMARK_OXIDATIVE_PHOSPHORYLATION, which at visit 1 is deregulated in the four studied experimental groups (i.e. iPD, GBA and G2019S *LRRK2* carriers, and prodromal cases). (**c**) Bar plot of LEG analysis in the shared HALLMARK_MYC_TARGETS_V2. Genes in this pathway, which are altered in all the four experimental groups, relate macromolecular synthesis linked to transcription and translation and nucleic acid metabolism. (**d**) Venn diagrams illustrating the relationship of the genes in the HALLMARK_MYC_TARGETS_V2, which at visit 1 is deregulated in the four studied experimental groups. (**h**) GSEA against GO-BP, KEGG, WikiPathways confirms results obtained against the Reactome database, indicating that deregulated processes largely relate to macromolecular synthesis linked to transcription and translation. (**e**) UpSet plot and (**f**) heatmap of the enriched GSEA against the Reactome compendium show the 13 shared pathways among the experimental groups, which were all down-regulated. (**g**) Shared LEG analysis of the 13 Reactome shared pathways largely identifies biological processes relative to translation and RNA metabolism. In the heatmaps, up- and down-regulated pathways are respectively in red and blue; p.adj<0.05. NES: normalized enrichment score. In GSEA, enrichment significance was assessed using a one-sided, permutation-based test on the NES, as implemented in the fgseaMultilevel() function, which employs an adaptive multilevel Monte Carlo algorithm and includes multiple comparison correction to estimate low p-values efficiently. In ORA (**a**, **c**, **g**, **i**), p-value was calculated with Fisher’s exact test and corrected for multiple hypotheses with the Benjamini-Hochberg procedure.

LEG analysis revealed that, in the HALLMARK_OXIDATIVE_PHOSPHORYLATION set, 28 genes were shared among the four experimental groups. Analysis against the GO-BP, which provides detailed gene descriptions in terms of function, participating processes and cellular location, indicated that these 28 genes were related to mitochondrial respiration (for example, aerobic electron transport chain (GO:0019646)), including complex I function (Extended Data Fig. 4a,b and Supplementary Table 7). This result is expected, given the role of mitochondria and complex I in the etiopathogenesis of PD. Meanwhile, shared processes in the HALLMARK_MYC_TARGETS_V2 set contained nine genes, which were mainly involved in macromolecular synthesis linked to transcription and translation and nucleic acid metabolism (Extended Data Fig. 4c,d and Supplementary Table 7). These alterations may point to genome instability because DNA damage is intrinsically associated with transcription and downstream translation^35,36^. Moreover, the linked transcription–translation system has been hypothesized to be perturbed in PD^37^, despite the highly rudimentary understanding of the role of these processes in the disease. Finally, a previous transcriptome analysis revealed alterations in ribosome-related pathways in the brains of patients with PD, and these alterations are also detectable in DNA repair-defective mice^16^.

To further confirm the alterations in macromolecular synthesis linked to transcription and translation in PD, we performed GSEA against the highly detailed Reactome compendium. The analysis confirmed our observations based on Hallmark: of the 466 identified deregulated pathways, only 13 were shared among the experimental groups and all were downregulated (Extended Data Fig. 4e and Supplementary Table 7). Two of these 13 pathways were related to mitochondrial function, whereas the remainder were associated with macromolecular synthesis linked to transcription and translation (Extended Data Fig. 4f). This is further confirmed by shared LEG analysis, which primarily identifies biological processes related to translation and RNA metabolism (Extended Data Fig. 4g and Supplementary Table 8).

GSEA against the KEGG, WikiPathways and GO-BP collections confirmed these findings, highlighting the downregulation of processes such as GOBP_RNA_PROCESSING, KEGG_RIBOSOME and WP_CYTOPLASMATIC_RIBOSOMAL_PROTEINS (Extended Data Fig. 4h).

### Patients with PD show dysregulation in DNA repair pathways

**Fig. 2:**
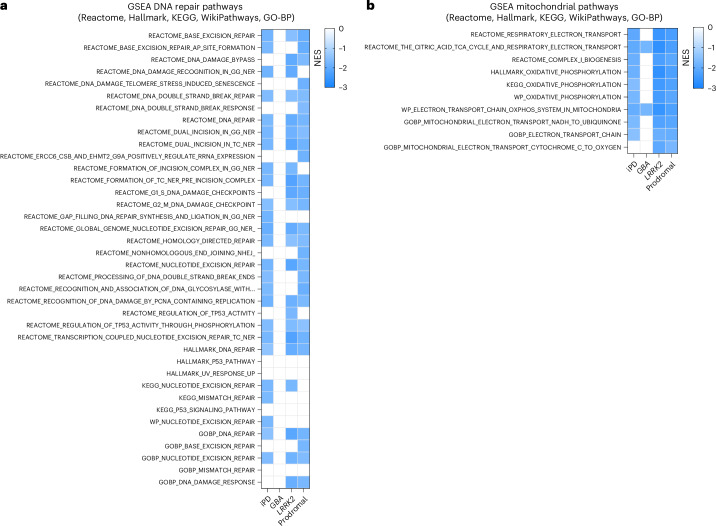
DNA repair pathways are perturbed in PD blood transcriptomic data. **a**, Heatmap showing downregulation at visit 1 of several DNA repair pathways against the five gene collections used in iPD, PD *LRRK2* G2019S carriers and prodromal cases. No changes in DNA repair pathways were detected in PD *GBA* carriers. **b**, Heatmap showing downregulation of mitochondrial pathways, which is expected given the central role of mitochondrial dysfunction in PD. In the heatmaps, all pathways are downregulated and represented in blue; white boxes indicate unchanged processes; *P*-adj < 0.05. In GSEA, enrichment significance was assessed using a one-sided, permutation-based test on the NES, as implemented in the fgseaMultilevel function, which uses an adaptive multilevel Monte Carlo algorithm and includes multiple comparison corrections to estimate low *P* values efficiently.

Based on our findings indicating alterations in mechanisms related to RNA biology, we specifically investigated whether DNA repair pathways are perturbed in PD and determined their upregulation or downregulation using GSEA. At visit 1, the analysis based on the Hallmark collection detected downregulation of DNA repair processes (that is, HALLMARK_DNA_REPAIR) in iPD, *LRRK2* and prodromal cases (Figs. 1b and 2a and Supplementary Table 5); the more detailed Reactome dataset revealed that downregulated processes included NER, consistent with our previous findings showing impaired NER capacity in patients with iPD and those with the *LRRK2* mutation^16^. By contrast, patients with *GBA* mutations showed relatively unaffected DNA repair pathways at visit 1. We validated these findings by performing GSEA against the KEGG, Hallmark, WikiPathways and GO-BP collections, all of which confirmed our observations in Reactome (Fig. 2a). Moreover, our analyses also detected downregulation of mitochondrial pathways (Fig. 2b), which is expected given the central role of mitochondrial dysfunction in PD.

To determine whether the observed changes in DNA repair pathways were influenced by medication, we analyzed the data while considering the dosages used by each patient, specifically the levodopa equivalent dose (LED)^38^, and the types of medications (see the combination of medications and the mean daily LED (LEDD) in Supplementary Table 1). In this analysis, we did not include patients with *GBA* mutations, who did not display the transcriptional DNA damage signature (Fig. 2a and Supplementary Table 3), or prodromal individuals, who did not take medications. Therefore, we focused on patients with iPD and *LRRK2* G2019S carriers. Medication variables were included as covariates in the regression analysis so that the model could account for their potential influence on the observed GSEA pathways. LEDD was treated as a continuous variable. Medication type was treated as a categorical variable, and regimens were divided into seven groups: (1) only levodopa/carbidopa; (2) only dopamine agonists; (3) levodopa/carbidopa and dopamine agonists; (4) levodopa/carbidopa and other medications; (5) levodopa/carbidopa, dopamine agonists and other medications; (6) other medications; and (7) no medications. When the medication field was left blank in the PPMI record, the patient was classified as not taking any medication (Supplementary Table 1). No patients were treated with dopamine agonists in combination with other medications. Other medications included A2A receptor antagonists, monoamine oxidase B (MAO-B) inhibitors, catechol-*O*-methyltransferase (COMT) inhibitors and anticholinergics.

**Extended Data Fig. 5:**
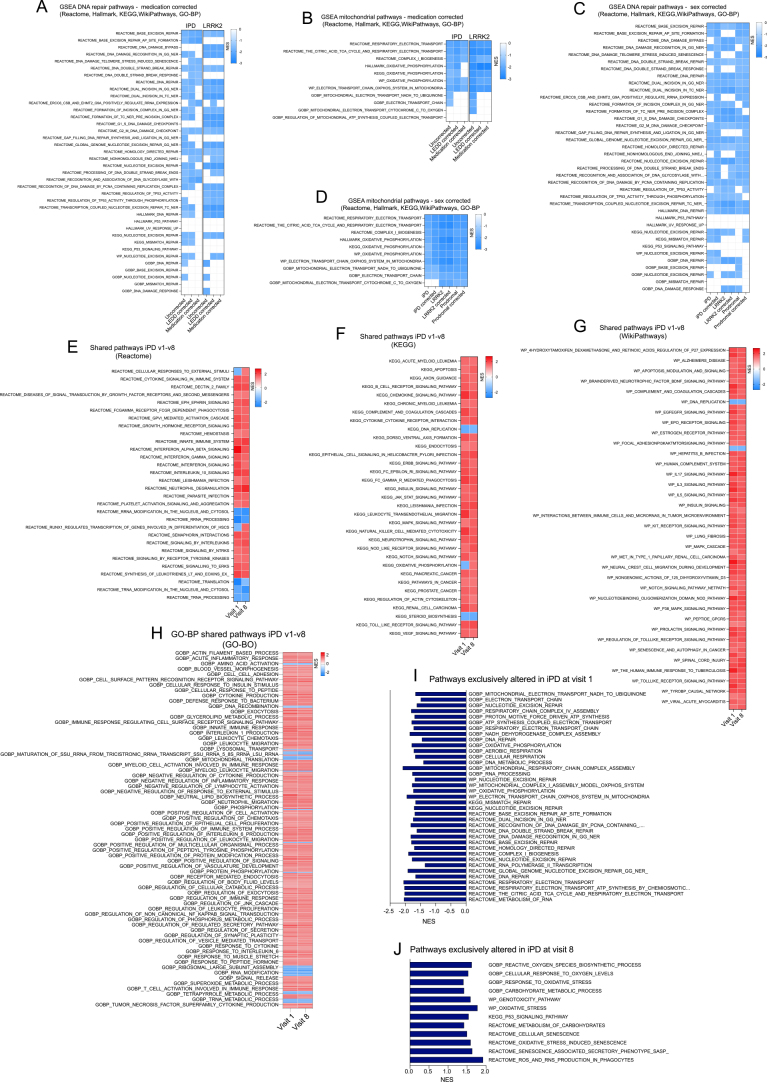
Deregulated Pathways in iPD between visit 1 and visit 8. (**a**) Alterations in DNA damage pathways in iPD and in PD *LRRK2* G2019S carriers persist after correction for LEDD and medication type. (**b**) The same applies to mitochondrial pathways. (**c**, **d**) Alterations in DNA damage and mitochondrial pathways in iPD and in PD *LRRK2* G2019S carriers persist also after correction for sex. (**e**-**h**). Heatmaps of GSEA identified altered pathways common to visit 1 and visit 8 based on (**e**) Reactome, (**f**) KEGG, (**g**) Wikipathways, and (**h**) GO-BP. Data confirm the findings obtained using the Hallmark database and shared altered pathways are consistent among the analyses. (**i**) Analysis against the four collections used shows that pathways deregulated exclusively at visit 1 are all down-regulated, consistently with Hallmark analysis, and relate to mitochondrial respiration and DNA repair. (**j**) Analysis against the four collections used shows that pathways deregulated exclusively at visit 8 in the two experimental groups with sufficient statistical power at this timepoint (i.e. iPD and GBA) are all up-regulated, consistently with Hallmark analysis, and relate to alterations in processes related to oxidant stress, carbohydrate metabolism, and genotoxic stress. In the heatmaps, up- and down-regulated pathways are respectively in red and blue, white boxes indicate unchanged processes; p.adj<0.05. NES: normalized enrichment score. In GSEA, enrichment significance was assessed using a one-sided, permutation-based test on the NES, as implemented in the fgseaMultilevel() function, which employs an adaptive multilevel Monte Carlo algorithm and includes multiple comparison correction to estimate low p-values efficiently.

GSEA revealed that changes in DNA damage-related pathways were generally unaffected by LED (Extended Data Fig. 5a). However, when correcting for medication type in iPD, significance was detected in 12 pathways against the 25 uncorrected or LEDD-corrected datasets. It is important to note that the correction for medication also resulted in the loss of significance in the KEGG and Hallmark oxidative phosphorylation pathways, which is inconsistent with PD pathogenesis considering the well-established role of mitochondrial dysfunction in the disease and the significant changes observed in other key mitochondrial pathways (Extended Data Fig. 5b). We also noted that the loss of statistically significant pathways in the correction analysis aligns with the intrinsic sample size reduction due to the introduction of additional variables in the analysis.

Data from patients with the *LRRK2* mutation were less affected by medication correction, and all downregulated pathways in the uncorrected analysis (REACTOME_ERCC_CSB…) were also altered after correction (Extended Data Fig. 5a). This result may indicate a more profound impact of DNA damage and repair in an *LRRK2* G2019S background, which is also consistent with our previous laboratory data obtained in fibroblasts^16^.

Collectively, these data show a modest impact of medication on alterations in DNA repair pathways. This result is also consistent with the observation that individuals with prodromal signs, who should not be taking PD medications, display similar changes in DNA repair pathways (Fig. 2a).

LEDD and medication type are highly correlated variables that interact with each other; therefore, they are considered confounding variables. Given these conditions, including all the variables in the same correction model may lead to issues involving multicollinearity, which hampers the accurate evaluation of their individual effects^39^. For this reason, we did not perform correction analysis simultaneously using both LEDD and medication type.

Finally, we also performed GSEA correcting for sex, given that PD differs between male and female patients. We found no impact on either DNA repair or mitochondrial pathways, which were also downregulated after correction (Extended Data Fig. 5c,d).

### Transcriptome alterations in PD evolve over time

**Fig. 3:**
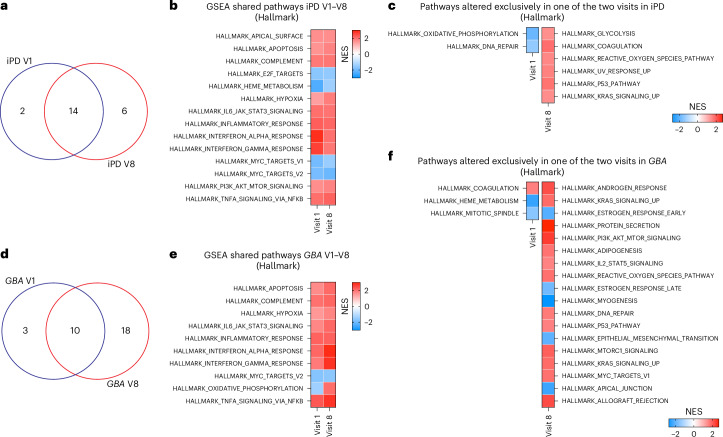
Evolution of differentially regulated pathways identified by GSEA during the 3-year observation period. **a**,**b**, Venn diagram (**a**) and heatmap (**b**) of enriched GSEA against the Hallmark set, revealing that, in iPD, 14 pathways were shared between visit 1 (V1) and visit 8 (V8). **c**, Heatmap illustrating the pathways that were exclusively altered in iPD at visit 1, all of which were downregulated and included DNA repair, and at visit 8, all of which were upregulated and included mechanisms related to oxidative stress, response to DNA damage and glycolysis. **d**,**e**, Venn diagram (**d**) and heatmap (**e**) indicating that, in PD *GBA* carriers, ten deregulated pathways were shared between visits 1 and 8; several of these deregulated processes involve inflammation. **f**, Heatmap illustrating the pathways that were exclusively altered in PD *GBA* carriers at visit 1 and visit 8. In the heatmaps, upregulated and downregulated pathways are represented in red and blue, respectively; *P*-adj < 0.05. Enrichment significance was assessed using a one-sided, permutation-based test on the NES, as implemented in the fgseaMultilevel function, which uses an adaptive multilevel Monte Carlo algorithm and includes multiple comparison corrections to estimate low *P* values efficiently.

We next leveraged the longitudinal construction of the PPMI cohort and compared the blood transcriptome at visit 1 and visit 8 (after 36 months) to determine whether expression changes may evolve during disease progression. We focused on groups with sufficient statistical power at both visits (that is, iPD and *GBA*) (Extended Data Fig. 2). GSEA against the Hallmark database revealed that, in iPD, the two time points shared several deregulated pathways (14 out of a total of 22) (Fig. 3a and Supplementary Table 5). Shared upregulated pathways were largely related to inflammation, whereas downregulated pathways were related to macromolecular synthesis linked to transcription and translation, as well as heme metabolism (Fig. 3b), which are coupled in blood cells^40^. Pathways that were exclusively altered at visit 1 were related to oxidative phosphorylation and, interestingly, to DNA repair, whereas pathways changed at visit 8 included mechanisms related to oxidative stress, response to DNA damage and glycolysis (Fig. 3c). In the *GBA* group, the proportion of shared deregulated pathways was smaller (that is, 10 out of a total of 31), and 18 pathways were exclusively deregulated at visit 8 (Fig. 3d). The higher number of visit 8-specific altered pathways, which points to more pronounced molecular shifts from physiological conditions during the observation period, may be consistent with the faster progression reported in patients with *GBA* mutations^41,42^. In the *GBA* group, shared pathways also included several processes related to inflammation, confirming the importance of inflammation in PD pathobiology (Fig. 3e). However, the pathways that were exclusively altered at visit 1 or visit 8 in the *GBA* group substantially differed from those in iPD. For instance, no alteration in DNA repair pathways was detected at visit 1, whereas upregulation, instead of downregulation, was observed at visit 8 (Fig. 3f). Interestingly, the HALLMARK_OXIDATIVE_PHOSPHORYLATION pathway, which was downregulated in iPD, was upregulated in the *GBA* group at visit 8, further suggesting different underlying pathogenic mechanisms in these PD subtypes (Fig. 3e).

GSEA analysis against other collections confirmed the findings obtained in iPD using the Hallmark database. Altered pathways shared between the two time points were consistent across all analyses (Extended Data Fig. 5e–h and Supplementary Table 5). When examining pathways altered exclusively at visit 1 or visit 8 using other collections, we observed changes that mirrored those found in the Hallmark database: pathways altered at visit 1 were all downregulated and related to mitochondrial respiration and DNA repair. At visit 8, the altered pathways were upregulated and associated with oxidative stress, carbohydrate metabolism and genotoxicity (Extended Data Fig. 5i,j and Supplementary Table 5). Importantly, at this time point, Reactome analysis also detected an increase in pathways related to senescence (Extended Data Fig. 5j), which is intrinsically associated with chronic DNA damage and oxidative stress^43^.

**Extended Data Fig. 6:**
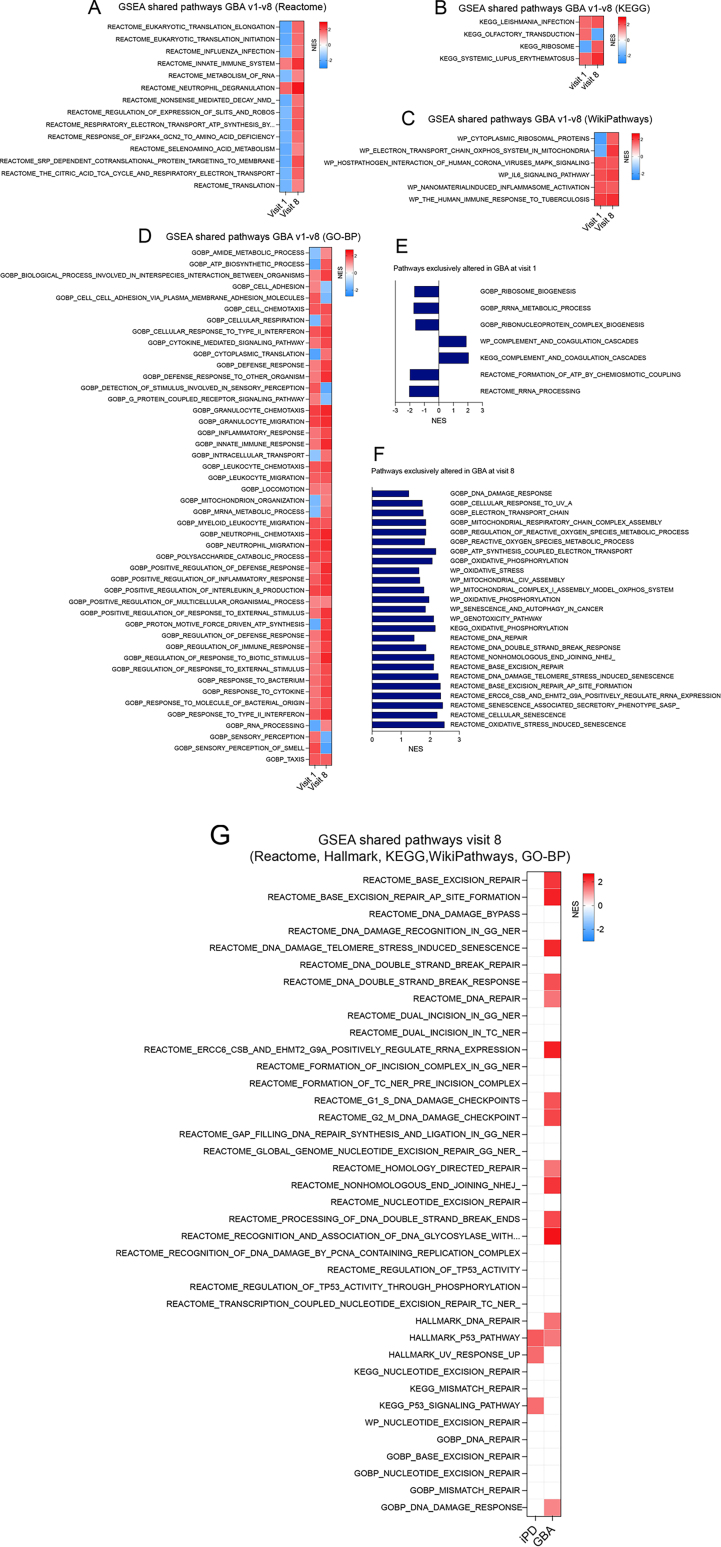
Deregulated Pathways in GBA cohort at visit 1 and 8. (**a-e**). Heatmaps of GSEA identified altered pathways common to visit 1 and visit 8 based on (**a**) Reactome, (**b**) KEGG, (**c**) Wikipathways, and (**d**) GO-BP. (**e**) Analysis against the four collections showing deregulated pathways in the GBA group exclusively at visit 1 and (**f**) visit 8. (**g**) GSEA in iPD and GBA patients focused on DNA repair pathways using the four collections reveals a different expression profile between visit 1 and visit 8.

In *GBA* specimens, analyses confirmed the downregulation of shared processes related to mitochondrial function and macromolecular synthesis linked to transcription and translation at visit 1, as well as their upregulation at visit 8; GSEA also detected a general upregulation of inflammation (Extended Data Fig. 6a–f). Increased oxidative stress and senescence were also detected in *GBA* specimens at visit 8 (Extended Data Fig. 6f), suggesting that these may be common downstream mechanisms.

Analysis with a specific focus on DNA repair pathways at visit 8 revealed a different scenario than at visit 1. At this later time point, all altered pathways were upregulated. In iPD, they were related to p53 and ultraviolet response (that is, HALLMARK_DNA_REPAIR and HALLMARK_P53_PATHWAY), which were unchanged at visit 1 (Fig. 2a); in *GBA*, they were related to several DNA repair processes (Extended Data Fig. 6g). These observations further substantiate the existence of differences between the iPD and *GBA* subtypes as well as time-dependent changes in the PD blood transcriptome.

Collectively, these observations confirm the involvement of DNA damage and nucleic acid metabolism in PD pathogenesis. Additionally, they unravel the time-dependent evolution of transcriptome alterations that differ between the iPD and *GBA* disease subtypes.

### Surrogate transcriptomic measures point to DNA damage accumulation in PD

We next sought to confirm defective genome maintenance in PD by inferring information from transcriptomic data. DNA damage is considered stochastic in nature; therefore, it is more likely to occur in longer genes than in shorter ones. Because DNA damage can block RNA polymerase progression, and therefore transcription, its accumulation is paralleled by a biased reduction in the transcripts of longer genes. Indeed, our laboratory and others have demonstrated that ALBATRO (analysis for length-biased alterations in transcription output) mirrors DNA damage accumulation^35,44,45^.

**Fig. 4:**
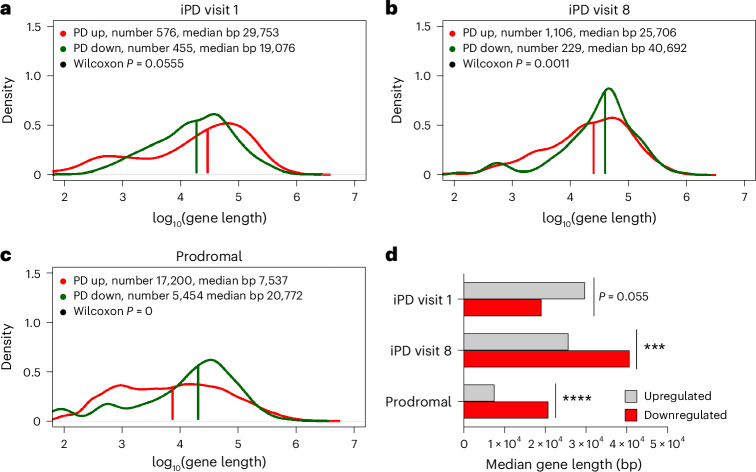
Biased reduction in the expression of longer genes in PD. **a**–**c**, Frequency plots of the lengths of upregulated and downregulated genes (red and green traces, respectively) in iPD at visit 1 (**a**), iPD at visit 8 (**b**) and prodromal cases (**c**). Gene length is presented on a log_10_ scale (iPD visit 1 *P* = 0.055, iPD visit 8 *P* = 0.0011, prodromal *P* = 0). **d**, Bar graph summarizing the findings in frequency plots, showing that downregulated genes were significantly longer in iPD at visit 8 and in prodromal cases. The *P* value for iPD at visit 1 is close to significance (*P* = 0.055). The statistical significance of the distributions was tested using a Wilcoxon two-sided signed-rank test (***P = 0.0011, ****P = 0).

To make ALBATRO informative, a sufficiently large number of DEGs must be identified. In this study, we arbitrarily set this number to be at least 100 genes per distribution. At visit 1, ALBATRO did not reveal any biased reduction in longer-gene transcripts in PD specimens compared to HC specimens: longer genes were even more represented in PD specimens, but the test was not statistically significant (median length of upregulated genes, 29,753 base pairs (bp); median length of downregulated genes, 19,076 bp; Wilcoxon *P* = 0.0555) (Fig. 4a,d). The overrepresentation of longer genes may reflect an adaptive protective response, given recent evidence indicating that longer transcripts are often related to antiaging genes^46^. However, at visit 8, ALBATRO detected significant downregulation of longer-gene transcripts in PD specimens (median length of upregulated genes, 25,706 bp; median length of downregulated genes, 40,692 bp; Wilcoxon *P* = 0.0011) (Fig. 4b,d). Interestingly, prodromal cases also showed biased downregulation of longer transcripts (Fig. 4c,d).

**Extended Data Fig. 7:**
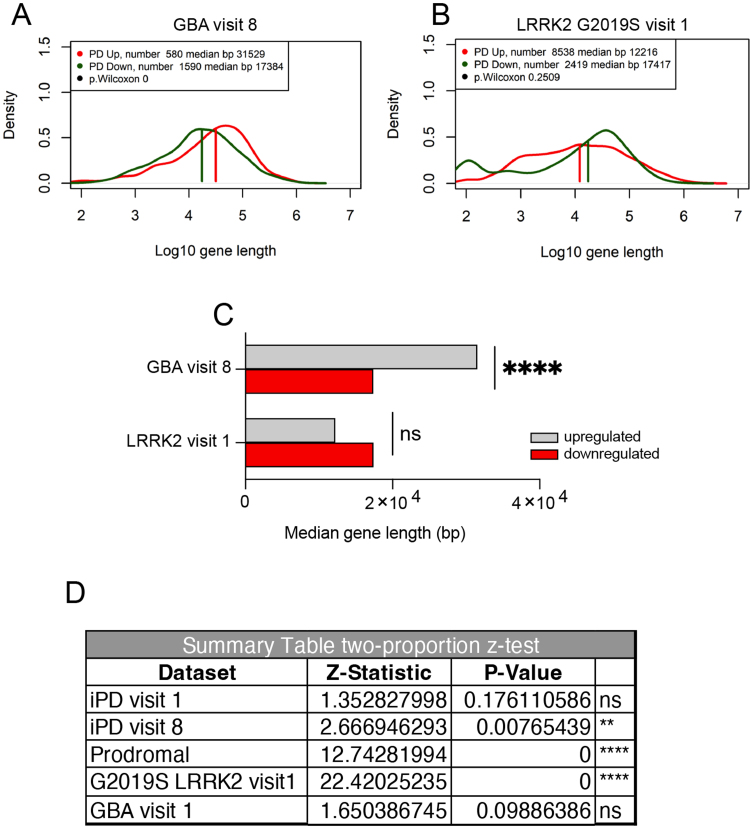
Analysis for length-biased alterations in transcription output in additional datasets. (**a, b**) Frequency plots of gene length of up- and downregulated genes (red and green trace respectively) in GBA patients at visit 8 and in G2019S *LRRK2* carriers at visit 1. (**c**) Bar graph showing statistically significant biased deregulation of short vs. long genes in GBA patients, in a opposite direction than that observed in iPD at visit 8 (p.Wilcoxon, p < 0.0001 ****). (**d**) Summary table of statistical test results. The table reports z-statistics and corresponding p-values for each comparison. (ns p>0.05, *p<0.05, ****p<0.0001)

ALBATRO detected slight effects in genetic *GBA* cases, and at visit 1, patients did not exhibit significant differences (data not shown). By contrast, at visit 8, *GBA* mutants displayed a statistically significant difference in the length of upregulated genes compared to downregulated genes, albeit in the opposite direction of that associated with DNA damage accumulation; that is, shorter genes were upregulated when compared to HCs (median length of upregulated genes, 31,529 bp; median length of downregulated genes, 17,384 bp; Wilcoxon *P* < 1 × 10^−07^) (Extended Data Fig. 7a,c). This evidence is consistent with our observation of upregulated DNA repair pathways in *GBA* specimens at visit 8 (Fig. 3f) and further supports the existence of different pathogenic mechanisms in *GBA* mutants. Meanwhile, patients carrying *LRRK2* G2019S at visit 1 showed a trend of downregulation of longer genes compared to shorter genes, but the test was not statistically significant (median length of upregulated genes, 12,216 bp; median length of downregulated genes, 17,417 bp; Wilcoxon *P* = 0.2509) (Extended Data Fig. 7b,c). Consistent with evidence obtained through GSEA, ALBATRO confirms similarities between the iPD and *LRRK2* groups.

ALBATRO is an indirect measure of DNA damage. Its outcome depends on the distinctive transcription profile at the time of analysis and may therefore pose specificity issues. To further substantiate that the PD transcriptomic profile may reflect DNA damage accumulation, we pursued a different, convergent approach focusing on genes located in common chromosomal fragile sites (CFSs). CFSs are known to be particularly vulnerable to DNA damage^47,48^ and have been organized in an open-source database (Supplementary Table 6)^49^. Therefore, we tested the hypothesis that, among DEGs in PD, the proportion of downregulated genes located in CFS regions might be higher than expected because of increased DNA damage hampering transcription. A two-proportion *z* test confirmed our hypothesis in *LRRK2* G2019S carriers at visit 1, in prodromal cases and in iPD cases at visit 8, but not in *GBA* carriers or patients with iPD at visit 1 (Extended Data Fig. 7d and Supplementary Table 6), fully aligning with evidence gained with ALBATRO.

### Transcriptional deregulation of DNA repair pathways shows a predictive association with disease severity

**Fig. 5:**
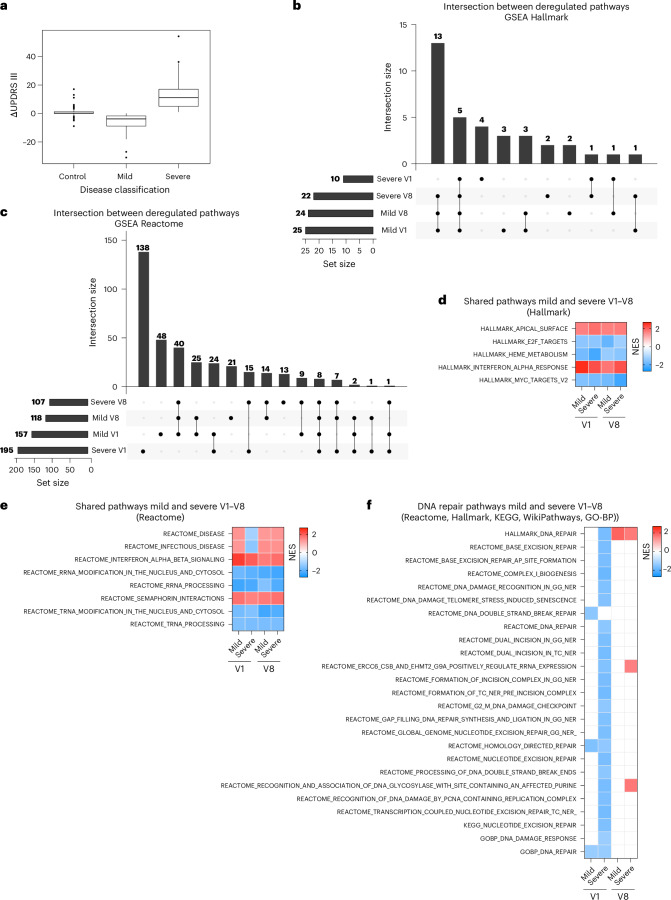
GSEA pathway analysis in patients with different disease progression states. **a**, Summary boxplot of ΔUPDRS III, defined as the difference in the UPDRS III score between visit 8 and visit 1, in patients with iPD. Boxes represent the interquartile range, from the 25th percentile (Q1) to the 75th percentile (Q3), with the center line indicating the median. Whiskers extend to 1.5 times the interquartile range from the quartiles; points outside the box represent outliers. Control individuals, *n* = 152; mild group patients (ΔUPDRS ≤ 1), *n* = 112; severe group patients (ΔUPDRS > 1), *n* = 226. **b**,**c**, UpSet plots illustrating the intersections of deregulated pathways in patients in the mild and severe groups over the 36-month observation period, as determined by GSEA against the Hallmark (**b**) and Reactome (**c**) datasets. **d**,**e**, Hallmark (**d**) and Reactome (**e**) heatmaps showing the deregulated pathways shared among mild and severe cases at both time points. Only five and eight shared pathways were identified by the Hallmark and Reactome GSEA, respectively. **f**, DNA repair pathways were predominantly downregulated in patients at visit 1 and in those who would display more severe deterioration of motor symptoms over the 36-month observation period. No major alterations in DNA repair pathways were detectable at visit 8. In the heatmaps, upregulated and downregulated pathways are represented in red and blue, respectively; white boxes indicate unchanged processes; *P*-adj < 0.05. In GSEA, enrichment significance was assessed using a one-sided, permutation-based test on the NES, as implemented in the fgseaMultilevel function, which uses an adaptive multilevel Monte Carlo algorithm and includes multiple comparison corrections to estimate low *P* values efficiently.

**Extended Data Fig. 8:**
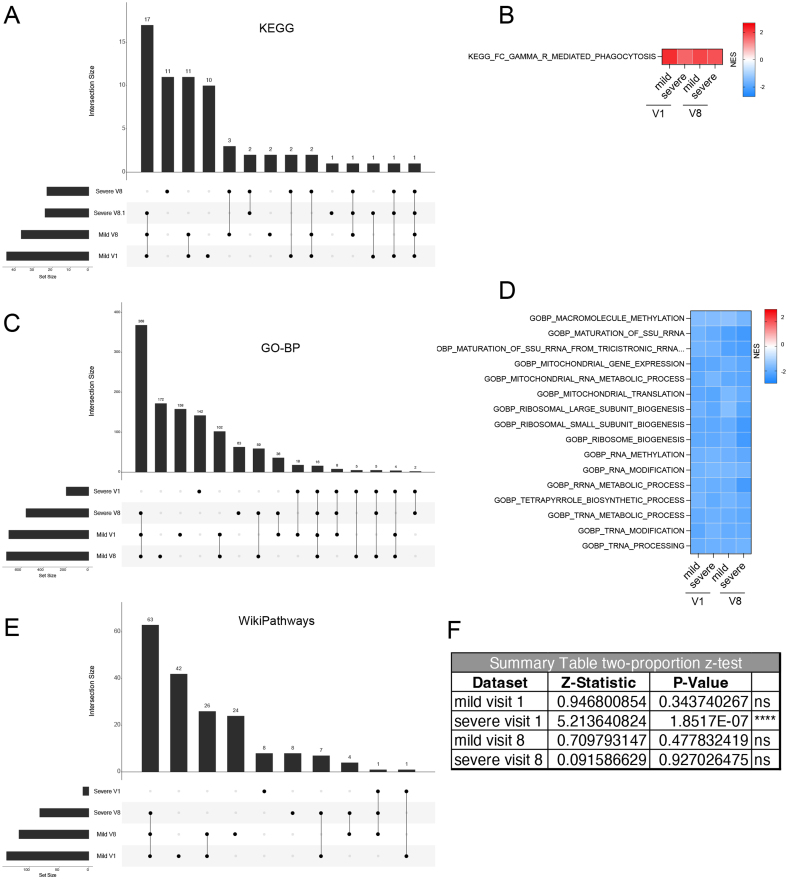
GSEA in iPD patients with different disease severity. (**a**) UpSet graph showing the interactions between deregulated pathways as determined by KEGG based GSEA in patients exhibiting mild versus severe disease progression during the three years period of observation. Only one altered pathway is shared among the experimental groups, it relates to inflammation, and (**b**) the heatmap shows that it is upregulated in all conditions. (**c**) UpSet graph showing the interactions between deregulated pathways as determined by GO-BP based GSEA in patients exhibiting mild versus severe disease progression during the three years period of observation. (**d**) 16 altered pathways are shared among the experimental groups, they relate to transcription, translation, and mitochondrial function, and the heatmap shows that they are downregulated in all conditions. (**e**) UpSet graph showing that no shared deregulated pathways are detected using GSEA against WikiPathways. (f) Summary table of statistical test results. The table reports z-statistics and corresponding p-values for each comparison. (ns p>0.05, **** p<0.0001).

Our results indicate that downregulation of DNA repair pathways occurs early in the symptomatic stage of PD (that is, detectable at visit 1) and evolves over time. Additionally, our data show that surrogate measures of DNA damage accumulation increase with disease progression. We next asked whether transcriptional deregulation of DNA repair pathways may have prognostic value and provide information on the rate of disease progression. Therefore, we stratified patients with iPD who were examined at both visits 1 and 8 into two groups with different progression rates. One group was composed of patients with increased severity as measured by the Movement Disorder Society’s Unified PD Rating Scale (UPDRS) III score (severe group; ΔUPDRS (UPDRS at visit 8 − UPDRS at visit 1) > 1, *n* = 226). Another group was composed of patients who did not display worsening of the UPDRS III score (mild group; ΔUPDRS ≤ 1, *n* = 112). We compared these groups to HCs (*n* = 152) (Fig. 5a). DEGs are listed in Supplementary Table 3. GSEA again revealed substantial heterogeneity; Hallmark- and Reactome-based analyses detected only five and eight shared pathways, respectively, between mild and severe cases at the two observed time points (Fig. 5b,c). Some of the identified processes were again inherent to RNA metabolism, whereas other pathways were related to disease and inflammation (Fig. 5d,e). Analyses based on KEGG and GO-BP confirmed these results (Extended Data Fig. 8a–d), whereas no shared pathways were detected using WikiPathways (Extended Data Fig. 8e).

When we investigated the expression of DNA repair pathways in these experimental groups, we found significant downregulation of various mechanisms only at visit 1 in those patients who displayed deterioration of motor symptoms at the follow-up visit after 36 months (visit 8) (Fig. 5f). We did not detect major alterations in DNA repair pathways at visit 8.

**Fig. 6:**
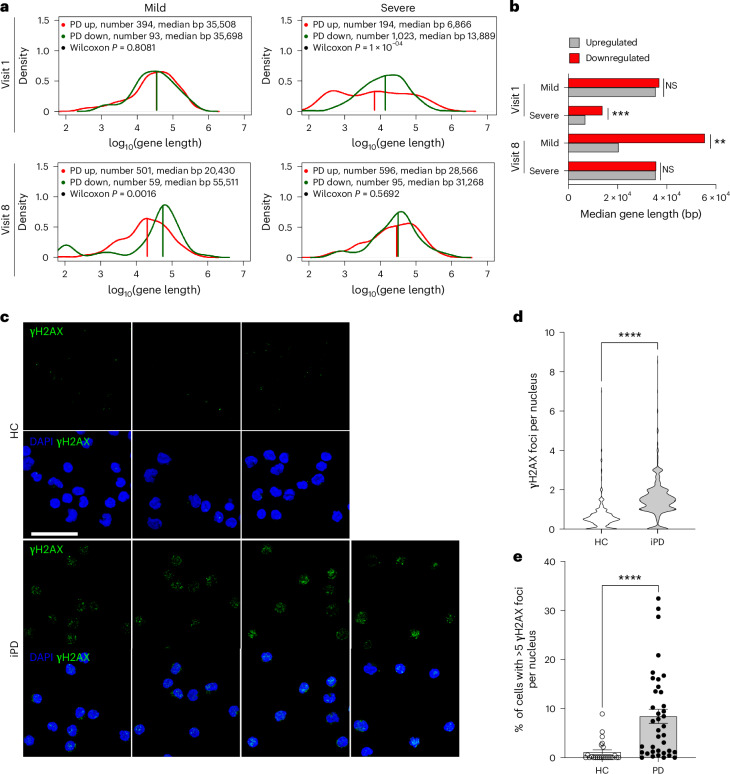
Surrogate measures of DNA damage in the PD blood transcriptome and PBMCs. **a**, Biased reduction in the expression of longer genes in patients with different disease progression states. Frequency plots of the lengths of upregulated and downregulated genes (red and green traces, respectively) are shown for patients with iPD who exhibited mild or severe progression at visits 1 and 8. Gene length is presented on a log_10_ scale. Mild group, visit 1, *P* = 0.8081; severe group, visit 1, *P* = 0.0001; mild group, visit 8, *P* = 0.0016; severe group, visit 8, *P* = 0.5692; two-sided Wilcoxon *P*). **b**, Bar graph summarizing the results shown in the frequency plots (two-sided Wilcoxon **P < 0.0016; ***P = 0.0001; NS, not significant). **c**, Representative immunohistochemistry images of γH2AX foci in PBMCs (patients with PD, *n* = 47; HC, *n* = 24). Scale bar, 50 μm. **d**, Violin plot showing the distribution of the number of foci per nucleus in PBMCs, indicating a higher signal in PD cells (PD, *n* = 772 cells; HC, *n* = 627 cells) (two-sided Mann–Whitney test, ****P < 0.0001). **e**, Graph showing an increased number of cells with more than five γH2AX foci per nucleus in PD PBMCs compared to the total counted cells (PD, *n* = 38; HC, *n* = 24). Data are represented as mean ± s.e.m. Each dot represents one individual (two-sided Mann–Whitney test, ****P < 0.0001).

When we analyzed surrogate measures of DNA damage accumulation at visit 1, ALBATRO detected reduced transcription of longer genes only in severe cases. This observation is logically consistent with the notion that defects in repair mechanisms, as evidenced at the transcriptional level, precede DNA damage accumulation. At visit 8, only mild cases displayed biased transcription reduction (Fig. 6a,b). Analysis of downregulated genes in common CFSs using a two-proportion *z* test at visit 1 detected a significant difference in severe cases but not in mild cases, aligning once again with the ALBATRO findings. Unlike ALBATRO, however, this approach failed to detect significant differences in mild cases at visit 8 (Extended Data Fig. 8f and Supplementary Table 6).

Collectively, these results indicate that measures of defective DNA repair and the accumulation of nuclear DNA damage, which were detectable only in severe PD cases at the earliest observation time point, have a predictive association with the disease course and therefore hold prognostic value.

### DNA damage surrogate measures in peripheral blood cells from patients with iPD

**Extended Data Fig. 9:**
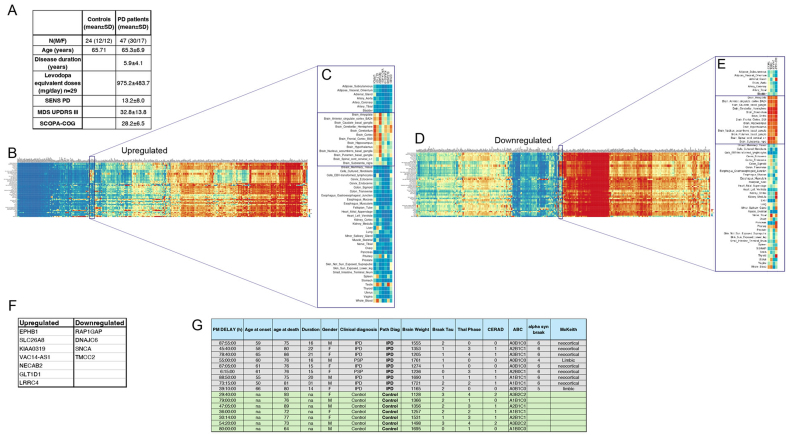
Demographic information on human samples used in the study. **a**) Table summarizing the characteristics iPD patients and healthy controls used in the analysis of DNA damage markers in PBMC; (**b**, **e**) Heatmaps originated with FUMA analysis from (**b**, **c**) up- and (**d**, **e**) downregulated genes highlighting sets of genes related to brain regions. (**f**) list of up- and downregulated genes inferred from FUMA heatmaps that are related to brain regions. (**g**) Table describing the characteristics of the Queen Square Brain Bank (QSBB) post-mortem substantia nigra pars compacta specimens (***Add path***: additional diagnosed pathologies, none of which were reported; ***Braak tau***: Braak staging based on tau pathology; ***Thal phase***: diffuse Aβ plaque burden; ***CERAD***: Consortium to Establish a Registry for Alzheimer’s Disease score; ***ABC***: tiered evaluation for 3 diffuse Aβ plaque and NFT burden, and neuritic plaque location and density based on CERAD score; ***alpha-syn Braak***: Braak staging based on alpha-synuclein pathology; ***McKeith***: McKeith consensus criteria for dementia with Lewy bodies diagnosis).

We next sought to perform in vivo validation of our bioinformatic findings in peripheral blood mononuclear cells (PBMCs) from patients with iPD. We monitored the basal levels (that is, in unstimulated cells) of phosphorylated histone H2AX (γH2AX) foci, which reflect DNA damage (Fig. 6c). PBMCs were obtained from a different cohort of patients with iPD that we characterized in previous reports (Extended Data Fig. 9a)^50,51^. As expected, patients with PD showed significantly higher numbers of γH2AX foci (PD mean = 1.487, s.d. = 0.899; HC mean = 0.595, s.d. = 0.996; *P* < 0.0001, Mann–Whitney test) (Fig. 6d) and cells with more than five foci per nucleus (PD mean = 11.76, s.d. = 7.001; HC mean = 0.624, s.d. = 0.4624; *P* < 0.0001, Mann–Whitney test) (Fig. 6e), thus confirming the bioinformatic findings.

### Alterations in peripheral cells involve genes participating in processes relevant to the PD brain

To provide additional evidence supporting the relevance of our findings in peripheral blood cells to the pathophysiology of the central nervous system, we used a bioinformatic workflow known as FUMA (functional mapping and annotation) analysis^52,53^. This tool allowed us to explore the associations between expression profile traits and cell specificity.

In essence, the tool disentangles the complexity of a bulk transcriptome, which includes multiple cell types, by comparing the results to expression patterns obtained at the single-cell level. FUMA thereby informs the representation of cell-specific molecular signatures within the pooled dataset. Ultimately, the approach yields a cross-tissue imputation of a transcriptional signature.

**Fig. 7:**
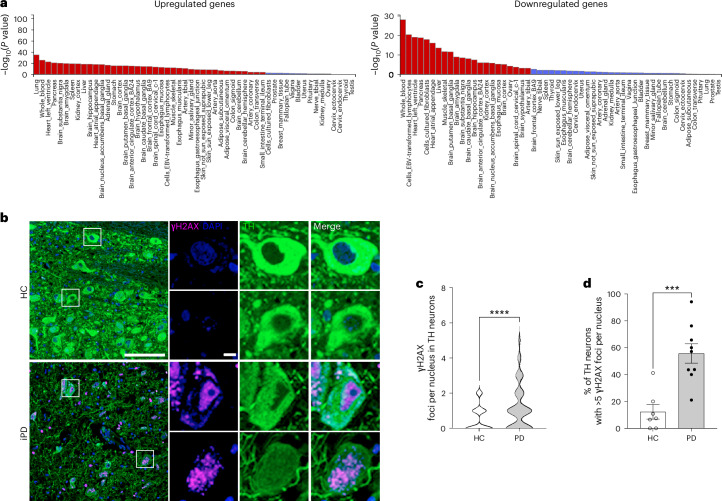
FUMA analysis of significant DEGs in iPD samples. The analytical framework indicates that the molecular signature includes a strong component of both upregulated and downregulated transcripts that are typical of cells in the putamen and basal ganglia of the brain. **a**, Histogram showing that several upregulated and downregulated genes in iPD blood also participate in brain and substantia nigra function (red bars indicate statistically significant associations, Bonferroni *P* < 0.05; https://fuma.ctglab.nl/). **b**, Representative immunohistochemistry images of TH-positive neurons in the substantia nigra pars compacta of patients with iPD (PD, *n* = 9; HC, *n* = 7), displaying γH2AX foci (purple). Scale bars, 100 μm, 20 μm. **c**,**d**, Quantification of the number of γH2AX foci in TH-positive neurons (**c**) and TH-positive neurons displaying more than five γH2AX foci (**d**), which demonstrate increased signs of DNA damage in iPD brains. Violin plot in **c** is showing the distribution of the number of foci per nucleus in TH-positive neurons. A total of 578 neurons from HCs and 314 neurons from patients with PD were analyzed (PD mean = 1.519, s.d. = 1.887; HC mean = 1.032, s.d. = 1.392; ****P < 0.0001, two-sided Mann–Whitney test). Each dot in **d** represents an individual. Bars represent mean ± s.e.m. (***P < 0.0007, two-sided Mann–Whitney test).

When interrogated about tissue-specific molecular signatures, FUMA analysis indicated, not surprisingly, that the most prominent contribution to the profile of DEGs came from the whole-blood signature. Of note, the analysis also highlighted the contribution of genes relevant to brain regions such as the putamen and the basal ganglia, which are central to PD pathogenesis (Fig. 7a). Heatmaps from FUMA analysis revealed a group of genes whose deregulation was particularly evident in gene sets related to brain regions (Extended Data Fig. 9b,e). These genes, which include the PD-related gene *SNCA*, are associated with GO classes that indicate processes relevant to neuronal function (for example, GO:0042982, amyloid precursor protein metabolic process; GO:0014069, postsynaptic density; GO:0030424, axon; GO:0042417, dopamine metabolic process; GO:0030517, negative regulation of axon extension; GO:0050804, modulation of chemical synaptic transmission) (Supplementary Table 9). This suggests that the alterations in DNA damage and repair observed in the blood of patients with PD may reflect similar defects in the brain.

We performed immunohistochemical measurements of the established DNA damage marker γH2AX in postmortem tissues from patients with PD (PD, *n* = 9; HC, *n* = 7; demographics in Extended Data Fig. 9d). Immunohistochemical analysis in the substantia nigra pars compacta revealed a significant increase in foci number in tyrosine hydroxylase (TH)-positive neurons (PD mean = 1.519, s.d. = 1.887; HC mean = 1.032, s.d. = 1.392; ****P < 0.0001, Mann–Whitney test) (Fig. 7b,c) as well as in the number of cells with more than five foci (PD mean = 12.13, s.d. = 6.475; HC mean = 6.857, s.d. = 2.067; ***P < 0.0007, Mann–Whitney test) (Fig. 7d). These findings are consistent with the evidence obtained at the bioinformatic level and, together with data on peripheral cells that we previously published^16^, strongly implicate defective DNA repair and DNA damage accumulation in PD pathogenesis.

### Validation in other datasets

**Fig. 8:**
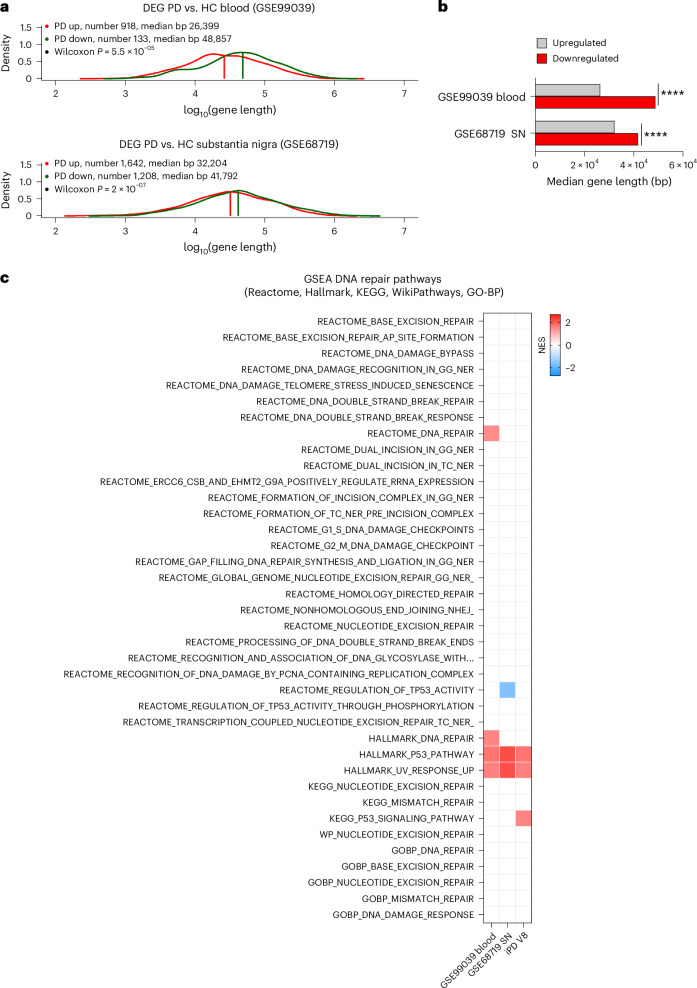
Alterations in DNA damage and repair measures in independent datasets. **a**, Frequency plots of the lengths of upregulated and downregulated genes (red and green traces, respectively) in the GSE99309 and GSE68719 datasets, which were derived from the blood and substantia nigra pars compacta, respectively (GSE99309, *P* = 0.000055; GSE68719*P* = 0.0000002). **b**, Bar graph illustrating biased suppression of longer genes in PD in both the blood and brain (two-sided Wilcoxon ****P < 0.0001). SN, substantia nigra. **c**, GSEA of the GSE99309 and GSE68719 datasets, which were generated from patients with a disease duration of >6 years, detected alterations that parallel those observed in iPD at visit 8. *P*-adj < 0.05. In GSEA, enrichment significance was assessed using a one-sided, permutation-based test on the NES, as implemented in the fgseaMultilevel function, which uses an adaptive multilevel Monte Carlo algorithm and includes multiple comparison corrections to estimate low *P* values efficiently.

Because bioinformatic analysis results may depend on the studied dataset, we sought to perform validation using other publicly available datasets. We applied ALBATRO to a dataset of PD blood transcriptome (GSE99039), which consists of 205 patients with iPD and 233 controls and has been reported to display reduced expression of *XRCC5* (ref. ^54^), a key gene in nonhomologous end joining. Additionally, we applied ALBATRO to a further dataset characterizing the transcriptome of the substantia nigra in 29 patients with PD compared to 44 neurologically normal controls (GSE68719)^55^. The analysis revealed a biased reduction in the levels of longer transcripts, as expected (Fig. 8a,b), thereby confirming our findings obtained in the PPMI cohort.

In these datasets, GSEA detected alterations that paralleled those observed in iPD at visit 8 (that is, 3 years after the intake visit) (Fig. 8c). This is expected, considering that the average age at sampling in GSE99039 was reported to be 62 ± 11 years compared to the age of onset of 56 ± 11 years (ref. ^54^), indicating that blood samples were collected after an average period of 6 years following the clinical diagnosis. Similarly, in GSE68719, the average disease duration at death was 7.6 ± 6.7 years (ref. ^55^). GO analysis of LEGs, identified using the Hallmark collection, confirmed alterations in DNA repair processes in both the GSE99039 and GSE68719 datasets (Supplementary Table 9).

In summary, analyses in independent datasets demonstrated the involvement of DNA damage and repair in PD pathogenesis, confirming our findings in the PPMI cohort.

## Discussion

Aging is a principal risk factor for PD, and the progressive accumulation of nuclear DNA damage is a causative hallmark of aging^10^. In this study, we aimed to expand previous evidence linking DNA damage and repair to PD^16^ by conducting a bioinformatic analysis of the blood transcriptome in the ‘state-of-the-art’ longitudinal PPMI cohort. Our results revealed a reduction in the expression of DNA repair pathways and a distinct pattern of DNA damage accumulation in PD. We confirmed our findings by analyzing independent cohorts and performing immunohistochemical studies on PBMCs and autopsy brain tissues from patients with PD. The observed effects were consistent between male and female individuals and were attributable to pathogenesis rather than to medications or age differences.

Defects in genome maintenance have been hypothesized, investigated and observed in several neurodegenerative disorders^11,56,57^. However, evidence gathered from patients with PD is limited^58^, particularly at the level of large longitudinal cohorts. Our study supports the involvement of age-related DNA damage and repair in PD, consistent with previous findings from our laboratories demonstrating reduced DNA repair capacity in fibroblasts from patients with PD and impaired dopaminergic systems in DNA repair-deficient mice^16^. These findings complement existing evidence indicating that α-synuclein activates the DNA damage response in vivo^17^ and that mitochondrial DNA damage accumulation participates in PD pathogenesis and may serve as a disease biomarker^59,60^. Our findings may also hold potential for providing prognostic biomarkers; longitudinal transcriptome analysis revealed that, at the intake visit (that is, visit 1), transcriptional derangement of DNA repair pathways and surrogate measures of DNA damage were exclusively observed in patients who exhibited a faster progression rate over a 3-year period.

Using a cutoff of log_2_(FC) >|0.322|, we detected relatively small changes in DEGs in patients with PD. This observation is consistent with previous studies analyzing the PPMI cohort, which used a cutoff of log_2_(FC) >|0.1| (refs. ^23,33^). Interestingly, we observed a higher number of DEGs in individuals with a prodromal status than in patients with PD, which also aligns with previous reports^23^ and a more severe effect in terms of transcriptome surrogate markers of DNA damage than in iPD. These effects may be explained by clinical evidence indicating that patients displaying prodromal signs represent a subgroup characterized by more severe progression^61^. Therefore, it could be hypothesized that, in prodromal individuals, the pathogenic cascade is fully underway and symptoms remain moderate because clinical manifestation requires cumulative damage over time. Notably, previous studies have shown molecular changes in prodromal stages that were not detectable in the symptomatic stages of neurodegenerative diseases^62^, along with more pronounced phenotypes based on some biochemical measures than in patients with PD^63^. This hypothesis is consistent with the chronic nature of PD and the potential role of accumulating macromolecular damage, such as DNA damage, in its development and progression. Our study further supports this idea, opening avenues for additional investigation. Moreover, alterations in gene expression during PD progression are nonlinear, with certain genes being more expressed in earlier phases than in later ones^33,64^.

The PD scientific community has been actively engaged in research efforts to develop body fluid and imaging biomarkers^65^. Recent studies have identified potential biomarker tests, such as the α-synuclein seed amplification assay^66^; one of the drawbacks of this approach is that it is currently performed on cerebrospinal fluid and has not yet been fully optimized for blood or other more accessible samples, making it an invasive test. Other studies successfully identified PD-specific blood transcriptome signatures in large cohorts comparable in size to the PPMI, but they did not examine patients longitudinally^54^. Additional research has analyzed the PPMI cohort but did not directly compare datasets from visits 1 and 8 to infer a correlation with motor symptoms^33^. Finally, other studies have revealed the potential of mitochondrial DNA damage as a PD biomarker^60,67,68^; this important evidence further substantiates the relevance of genome instability in PD, thereby strengthening our results. Our findings complement those described in these important studies and may contribute to the development of a reliable PD biomarker panel.

We detected a notable difference in the transcriptomic landscape between slow and fast PD progressors. In the latter group, we observed significant transcriptome changes over the 3-year observation period. It is tempting to speculate that the reduced genome maintenance observed in fast-progressing patients at visit 1 contributes to this transcriptome evolution, paralleling what is observed in cancer as a result of genome instability^69^. However, it is conceivable that this phenomenon may occur at a slower pace in PD. Indeed, studying mouse models of genome instability, we found that mild rather than pronounced DNA repair defects elicit a dopaminergic phenotype that recapitulates PD, also due to a pronounced adaptive, antioxidant response elicited by severe transcription-stalling DNA damage^16,45^. Interestingly, our data indicate that the suppression of DNA repair pathways is reversed at more advanced stages of pathogenesis when surrogate measures of DNA damage instead appear. This evidence points to an interplay between the kinetics of DNA damage accumulation and the activation of repair mechanisms. Here, the progressive accumulation of DNA damage during pathogenesis may stimulate the activation of DNA repair pathways, which are initially downregulated. This possibility is consistent with our previously published observations indicating that chronic α-synuclein stress activates the DNA damage response^17^. Further focused studies will be necessary to better understand the evolution of the transcription landscape in PD, its implications, and its connections with DNA damage and repair mechanisms.

We did not detect any alterations related to DNA repair pathways in patients with *GBA* mutations, an observation that may suggest distinct pathogenic mechanisms in this specific group of patients with PD. This possibility would also be consistent with the reported differences in disease presentation and age of onset among *GBA* carriers^70^.

Stratification of patients is crucial in the field of neurodegenerative diseases^71^. Patient heterogeneity can be catastrophic to the success of clinical trials because therapeutic interventions may have different effects depending on the individual pathogenic mechanisms driving disease progression. Our study provides evidence that defective DNA repair pathways can serve as predictive markers for the severity of motor symptoms and the rate of disease progression. Implementing this screening test during patient recruitment for future trials could help refine patient selection, reduce heterogeneity in study cohorts and design more informative clinical trials^6,8^.

Our findings in peripheral tissues have implications for the central nervous system. FUMA analysis revealed that transcripts critical for brain function significantly contributed to the list of DEGs. Furthermore, neuropathological analysis of postmortem brains detected increased markers of DNA damage in PD. Overall, our study substantiates the concept that peripheral tissues can provide insights into central pathology and aligns with evidence showing that risk loci for PD affect various cellular processes that are not necessarily confined to the brain, one of its anatomical areas or a specific cell type^72^.

DNA occupies the highest hierarchical position in biological information storage and processing, and any compromise in its fidelity has broad impacts. DNA damage accumulation has been associated with key mechanisms of neurodegeneration, including mitochondrial defects, oxidative stress, proteotoxic stress and inflammation^73–77^. Therefore, it is plausible that the defective DNA maintenance in PD interacts with other well-established pathogenic mechanisms. For instance, reduced NER capacity sensitizes cells to MPTP (1-methyl-4-phenyl-1,2,3,6-tetrahydropyridine) and triggers α-synuclein stress, while *LRRK2* biology influences mitochondrial DNA quality^16,68^. Further focused studies are necessary to explore the mutual interactions and potential synergies between genome stability and PD pathogenic mechanisms.

From a technical standpoint, a biased reduction in the expression of long genes serves as a reliable surrogate marker of DNA damage accumulation^35,44,45^. The detection of DNA chemical modifications can be challenging due to the wide variety of possible alterations, their rapid kinetics and their diverse effects on cellular function^78,79^. These factors may lead to artifactual measures; consequently, attention has been devoted to the use of alternative systems, such as those based on measures of repair efficiency^80^. Biased reduction in long-gene expression circumvents these limitations by providing a measure of age-related DNA damage accumulation^35^.

Our study also has some limitations that must be addressed in future dedicated investigations. For instance, blood cell populations undergo alterations in PD^63^, and hematopoiesis is accelerated during disease progression^81^. These processes could influence the analyses we performed, and differences in DNA repair may, to some extent, reflect blood cellular changes intrinsically associated with PD. This limitation could be circumvented, at least in part, by taking advantage of deconvolution methods^82^.

The main goal of this study was to identify a fingerprint of altered DNA maintenance in the PPMI cohort. We successfully detected these alterations in patients who exhibited faster progression of motor symptoms over a 36-month observation period. Defects in DNA repair pathways may serve as predictive criteria for disease progression rates. Furthermore, our analysis revealed differences in the transcriptional landscape between slow and fast progressors, shedding light on potential variations in the underlying pathogenic mechanisms. Collectively, our study identifies a biomarker with a predictive association with PD severity, reveals new pathogenic mechanisms and demonstrates the evolution of the PD blood transcriptome during disease progression. While further validation in other independent cohorts will be necessary to achieve conclusive results and translate our findings into clinical practice, our study lays the foundation for developing future tools to predict PD progression, improve therapy planning and stratify patients in clinical trials.

## Methods

### Ethics statement

Brain samples were provided by the Queen Square Brain Bank for Neurological Disorders and were regulated by a material transfer agreement (MTA #10-2019), which ensured that the samples were obtained and used in accordance with legal and ethical requirements.

The publicly available transcriptome data from the PPMI cohort have been used in accordance with the repository’s guidelines and policies.

PBMCs from patients with iPD were obtained from the Profiling PD study. The study was approved by the medical ethics committee of the Leiden University Medical Center, and written informed consent was obtained from all patients with PD.

### PPMI data retrieval and preprocessing, quality control and batch correction

Blood transcriptome data from the PPMI cohort were downloaded from https://ida.loni.usc.edu/pages/access/geneticData.jsp#441. Briefly, the data were downloaded on 11 May 2022. Downloaded Salmon files were imported into R using tximport (version 1.26.1). Statistical power analysis was performed using the pwr package. Only transcripts with at least ten reads in total were retained for analysis. Raw read counts were supplied to DESeq2 (version 1.40.2), which was used to perform differential expression analysis across PD groups and controls. Data quality was assessed by identifying the distribution of gene counts, the library size, the clustering in a PCA, and the ability to distinguish expected DEGs between female and male individuals. To reduce noise without the need for arbitrary filters on low-count genes, the ‘apeglm’ v1.20.0 shrinkage estimator was applied^83^.

To adjust for medication effects, we included medication variables as covariates in the DESeq2 design. LEDD was included as an independent variable so that the model could account for the potential influence of the medications on the observed GSEA pathways. Medication type was treated as a categorical variable. We stratified patients into seven classes based on the medications used: (1) only levodopa/carbidopa; (2) only dopamine agonists; (3) levodopa/carbidopa and dopamine agonists; (4) levodopa/carbidopa and other medications; (5) levodopa/carbidopa, dopamine agonists and other medications; (6) other medications; and (7) no medications. When the medication field was left blank in the PPMI record, the patient was classified as not taking any medication. No patients took dopamine agonists in combination with other medications. Other medications included A2A receptor antagonists, MAO-B inhibitors, COMT inhibitors and anticholinergics.

Analyses were conducted on 53 of the 58 prodromal individuals who remained clinically stable and did not develop PD symptoms within 2 years, as documented in the PPMI database. The remaining five cases, for which no information was available, were excluded from the study.

The rlog transformation was applied to the normalized counts to improve the distances and clustering for the PCA. To identify low-quality samples, we performed a visual inspection of the distribution of the reads.

### Identification of DEGs

We focused our investigations on two time points: baseline (that is, the very first visit) and visit 8 (that is, 36 months after the first visit). DEGs were estimated using log_2_(FC) analysis, the Wald test and false discovery rate (FDR) *P*-value correction as implemented in DESeq2. A gene was defined as differentially expressed when its FDR was <0.05.

### Identification of genetic variants in patients with PD

Patients with PD, control individuals, and those with known genetic mutations associated with PD, such as *GBA*, *LRRK2* and *SNCA*, were enrolled in the genetic cohort; all information can be found on www.ppmi-info.org. For this study, we selected only patients with a diagnosis of PD and unaffected control individuals, with or without mutations in *LRRK2* G2019S or *GBA* (*GBA* N370S, *GBA* T408M, *GBA* E365K, *GBA* IVS2, *GBA* 84GG or *GBA* L444P). Demographic data are listed in Supplementary Table 1.

### Pathway analysis

Pathway enrichment analysis was conducted using two different approaches: ORA^24^ and GSEA^34^. ORA focuses on significant DEGs (FDR ≤ 0.05) to determine whether these genes overrepresent known biological functions or processes. Because ORA processes only significant DEGs, it provides information based on relatively large differences. However, functional differences could stem from small, coordinated changes in groups of related genes (that, is expression modules), which, by construction, cannot be detected by a method limited to significant DEGs. GSEA addresses this limitation by analyzing all genes ranked according to the differences in expression between two groups.

ORA was performed using the Enrichr web tool (version 3.2) and the GO-BP 2018 database^84–86^ on the prefiltered list of DEGs obtained as described in the previous section. Fisher’s exact test was performed to determine the likelihood of obtaining at least the equivalent number of genes as those that actually overlap between the input gene set and the genes present in each identified pathway.

GSEA was conducted on an unfiltered ranked list of genes. Genes in each PD group compared to controls were ranked by the level of differential expression using a signal-to-noise metric and a weighted enrichment statistic according to the formula res1$stat=log10(res1$pvalue)/sign(res1$log2FoldChange) in the R language context^34^. The statistical significance of the pathway enrichment score was ascertained by permutation testing over size-matched random gene sets. Multiple testing was controlled for false positives with a family-wise error rate threshold of 5% (ref. ^34^), which is statistically more conservative than the FDR.

We used GSEA (version 4.2.2), which includes the KEGG database, the Reactome Pathway Database, the Hallmark Gene Set Collection (https://www.gsea-msigdb.org/gsea/msigdb/collections.jsp) and WikiPathways (https://www.wikipathways.org/).

Pathway information was obtained from the KEGG database available at MSigDB (https://www.broadinstitute.org/gsea/msigdb/index.jsp) or from the Hallmark Gene Set Collection (https://www.gsea-msigdb.org/gsea/msigdb/collections.jsp).

### Gene length and CFS analysis

BioMart (version 3.17)^87^ was used to retrieve the gene length (exons and introns calculated as gene length = (gene_end – gene_start)) for all genes in the human genome. Each list of DEGs was divided into downregulated and upregulated genes. The distribution of the log_10_(length) of upregulated and downregulated genes was evaluated for normality using the Shapiro–Wilk test. Because all distributions were not normal, a Mann–Whitney Wilcoxon test for unpaired samples was used to evaluate whether the distributions of gene lengths of upregulated and downregulated DEGs differed across the different comparisons. Additionally, a relative frequency (kernel density) plot of gene length and probability density for DEGs in each comparison was created using the density function implemented in R. Kernel density estimates are related to histograms, but with the possibility of smoothing and continuity by using a kernel function. The *y* axis represents the density probability for a specific range of values on the *x* axis. The list of CFSs was downloaded from https://webs.iiitd.edu.in/raghava/humcfs/ (HumCFS: a database of human CFSs^49^) (Supplementary Table 6). A two-proportion *z* test was performed to determine whether the proportions of upregulated versus downregulated genes in CFSs are different using an *α* level of 0.05.

### ΔUPDRS classification of patients

ΔUPDRS was calculated as the UPDRS III score at the last visit minus the UPDRS III score at the first visit.

### Gene expression analysis with FUMA

FUMA is an online tool that identifies sets of significantly upregulated or downregulated DEGs across human tissue types with a Bonferroni-corrected *P* value of <0.05. GENE2FUNC, a tool of FUMA (https://fuma.ctglab.nl/) v1.5.4, was run using GTEx (Genotype–Tissue Expression) v8 tissue types and general tissue types^52^.

An enrichment analysis of DEGs in different tissues was carried out. The direction of expression was considered with *P* < 0.1. Tissue specificity was tested using hypergeometric tests. FUMA reports gene sets with a *P*-adj value of ≤0.05 and the number of genes that overlap with the gene set >1 by default. Data are reported in a histogram representing the −log_10_(*P* value) with red (significant) and blue (not significant) bars.

### Detection of γH2AX foci in PD PBMCs

Frozen PBMCs were thawed and allowed to recover in RPMI medium containing 10% FBS for 1 h at 37 °C. The experimental setup included a positive control group in which PBMCs were irradiated at 2 Gy using the Xstrahl RS320 X-ray irradiator (data not shown). Samples were incubated for 1 h at 37 °C, centrifuged and smeared on a glass slide. After air-drying for 10 min at room temperature, PBMCs were fixed with 4% paraformaldehyde, permeabilized using 0.1% Triton X-100 and blocked with 3% BSA in PBS overnight at 4 °C. Samples were incubated with γH2AX antibody (1:1,000, 05-636, Merck) in blocking solution containing 0.05% Tween-20 for 90 min at room temperature, washed with PBS-T containing 1.5% BSA and incubated with a secondary donkey anti-mouse Alexa 488 antibody (1:800, A-21202, Invitrogen) for 45 min at room temperature. Nuclei were stained using Hoechst 33342 (1:1,000 in PBS, H3570, Thermo Fisher) and were mounted with Aqua-Poly/Mount (18606-20, Polysciences). Samples were processed and imaged at Erasmus MC using a Leica LSM700 confocal microscope. The images were sent in a blinded manner to the IFOM laboratory in Milan, where they were analyzed in a semiautomated way using a macro in Fiji. The results did not pass the normality test, as indicated by the D’Agostino and Pearson, Anderson–Darling and Shapiro–Wilk tests; therefore, significance was calculated using the Mann–Whitney test.

### Immunostaining of tissues

Formalin-fixed, paraffin-embedded and anonymous human midbrain tissue sections derived from patients with PD and age-matched HCs were kindly provided by the Queen Square Brain Bank for Neurological Disorders.

To minimize autofluorescence, sections were treated for photobleaching as previously described^88^. Briefly, samples were incubated in alkaline hydrogen peroxide solution (25 ml PBS, 4.5 ml H_2_O_2_ 30%, 0.8 ml NaOH 1 M) in clear plastic petri dishes and exposed to white light by sandwiching the immersed slides between two light-emitting-diode panels for 45 min at 4 °C (refs. ^88,89^). The procedure was repeated twice, with full solution change between the processes. Sections were then rinsed in TBS, pH 7.5, containing 0.01% Tween-20 and 100 mM sucrose (TBS-Ts) and blocked for nonspecific binding with 5% donkey serum in antibody dilution buffer (2% BSA, trehalose 1 mM in TBS). Anti-TH (1:500, MAB 318, Merck Millipore) and anti-γH2AX (1:500, ab11174, Abcam) were used diluted in antibody dilution buffer overnight at 4 °C. After washes in TBS-Ts, sections were incubated with Alexa Fluor antibodies (1:250 in antibody dilution buffer, Thermo Fisher) and DAPI (1:3,000 in TBS-Ts). Finally, ProLong Glass antifade medium (P36980, Thermo Fisher) was used as the mounting medium. Sections were processed in a single batch to prevent batch effects. Images were acquired with the Leica SP8 STED (stimulated emission depletion) imaging acquisition system at 40× magnification, using the same initial parameters set on a control slide; each image was composed of 40 tiles on average to cover a large area and ten z-stack steps of 0.5 μm. Foci were counted over a three-dimensional z-stack in a semiautomatic way using a script in Fiji/ImageJ software v1.54. We analyzed a total of 578 and 314 neurons from HCs and patients with PD, respectively. We tested the data for normality using the D’Agostino and Pearson, Anderson–Darling, Shapiro–Wilk and Kolmogorov–Smirnov tests (GraphPad v9), none of which detected a normal distribution. Therefore, statistical significance was assessed using a Mann–Whitney test.

### Statistics and reproducibility

All statistical analyses were conducted using R v4.3.1 or GraphPad Prism v9. Sample sizes were determined by dataset availability and validated using retrospective power analysis (pwr package v1.3-0) in RStudio, confirming sufficient power to detect transcriptomic differences between the compared groups. Experimental groups with effect sizes <0.6 were excluded due to low statistical power. Data normality was tested using the D’Agostino and Pearson, Anderson–Darling, Shapiro–Wilk and Kolmogorov–Smirnov tests, and subsequent statistical tests were selected accordingly.

Differential gene expression was analyzed using DESeq2 v1.40.2, applying the Wald test and the Benjamini–Hochberg procedure to control the FDR (FDR < 0.05). A log_2_(FC) threshold of |0.322| (1.25-fold) was used throughout the study. Pathway enrichment was performed using both ORA and GSEA across multiple gene set databases (Hallmark, Reactome, KEGG, WikiPathways, GO-BP), with significance thresholds set at *P*-adj < 0.05 (FDR). Age, sex, medication type and LED were used as covariates in the DESeq2 model.

We performed GSEA using the ‘fgseaMultilevel’ function from the fgsea R package, which implements a one-sided statistical test for enrichment. This approach separately evaluates whether a given gene set is significantly enriched among the most upregulated or most downregulated genes, based on the sign and magnitude of the normalized enrichment score (NES). A one-sided test is appropriate in the GSEA framework because gene sets typically exhibit directional biological effects—that is, upregulation and downregulation often imply distinct regulatory or functional consequences. Using a two-sided test would conflate these effects and reduce interpretability, particularly in contexts such as this study, where understanding pathway activation versus repression is critical. The NES and associated *P* values were computed separately for positive and negative enrichment, allowing clear and statistically sound distinction between upregulated and downregulated pathways. Of note, this directional approach is consistent with the original GSEA methodology and standard practice in transcriptomic pathway analysis.

For gene length analysis, we tested the normality of the distribution using RStudio and found that the data did not follow a normal distribution (*P* < 0.05 in the Shapiro test). Thus, we performed analyses using a nonparametric test (Wilcoxon signed-rank test). Fragile site enrichment was tested using a two-proportion *z* test; in this case, normality was also assessed using the Shapiro–Wilk test. γH2AX foci in PBMCs and postmortem brain tissues were quantified in blinded experiments using semiautomated image analysis.

Results were validated in independent transcriptomic datasets (GSE99039 and GSE68719) and confirmed by converging statistical and biological evidence.

### Reporting summary

Further information on research design is available in the Nature Portfolio Reporting Summary linked to this article.

## Supplementary information

Reporting Summary

Supplementary Table 1 Demographic table.

Supplementary Table 2 List of patients from the PPMI dataset.

Supplementary Table 3 Differentially expressed genes list.

Supplementary Table 4 Deregulated pathways inferred by ORA.

Supplementary Table 5 Deregulated pathways inferred by GSEA.

Supplementary Table 6 Common chromosomal fragile sites.

Supplementary Table 7 LEG analysis shared pathways visit 1 – Hallmark.

Supplementary Table 8 LEG analysis shared pathways visit 1 – Reactome.

Supplementary Table 9 LEG analysis GSE 99039 and GSE 68719 datasets.

Supplementary Table 10 Index of Supplementary Tables.

## Data Availability

Raw data for blood transcriptome analysis and metadata used in this study are available for download from the Parkinson’s Progression Markers Initiative (PPMI) database. PPMI data were initially downloaded from https://ida.loni.usc.edu/pages/access/geneticData.jsp#441 on 11 May 2022 after completing a data user agreement. Salmon files are available on the LONI IDA website (https://ida.loni.usc.edu/pages/access/geneticData.jsp#441) upon formal request with a data use agreement. Patient information (clinical scale assessment, DaTscan, magnetic resonance imaging, genome sequencing data, patient history and medications) is also available on the LONI IDA website.

## Code availability

All code used for the study is available via Github at https://github.com/DNAdamageinPD/Natureageing, via Zenodo at 10.5281/zenodo.16728586 (ref. ^90^) and via Code Ocean at 10.24433/CO.1403558.v2.
