# Author Correction: A blood-based DNA damage signature in patients with Parkinson’s disease is associated with disease progression

**DOI:** 10.1038/s43587-025-01037-3

**Published:** 2025-11-19

**Authors:** Daisy Sproviero, César Payán-Gómez, Chiara Milanese, Sander Barnhoorn, Shixiang Sun, Akos Gyenis, Domenico Delia, Tammaryn Lashley, Jan H. J. Hoeijmakers, Jan Vijg, Pier G. Mastroberardino

**Affiliations:** 1 IFOM-ETS, The AIRC Institute of Molecular Oncology, Milan, Italy; 2 Universidad Nacional de Colombia, Sede de La Paz, La Paz, Colombia; 3 Departmental Faculty of Medicine and Surgery, UniCamillus-Saint Camillus International University of Health and Medical Sciences, Rome, Italy; 4 Department of Molecular Genetics, Erasmus MC, Rotterdam, the Netherlands; 5 Department of Genetics, Albert Einstein College of Medicine, Bronx, NY USA; 6 Institute for Genome Stability in Ageing and Disease, Cluster of Excellence for Aging Research, Faculty of Medicine, University of Cologne, Cologne, Germany; 7 The Queen Square Brain Bank for Neurological Disorders, Department of Clinical and Movement Neuroscience, UCL Queen Square Institute of Neurology, London, UK; 8 Department of Neurodegenerative Diseases, UCL Queen Square Institute of Neurology, London, UK; 9 Princess Maxima Center for Pediatric Oncology, Oncode Institute, Utrecht, the Netherlands; 10 Center for Single-Cell Omics, School of Public Health, Shanghai Jiao Tong University School of Medicine, Shanghai, China; 11 Universita’ degli Studi dell’Aquila, L’Aquila, Italy

**Keywords:** Parkinson's disease, Predictive markers, Ageing

Correction to: *Nature Aging* 10.1038/s43587-025-00926-x, published online 5 September 2025.

In the version of the article initially published, in the “Transcriptional deregulation of DNA repair pathways shows a predictive association with disease severity” section, the text “ΔUPDRS (UPDRS at visit 8 − UPDRS at visit 1) > 0, *n* = 226)” should have read “ΔUPDRS (UPDRS at visit 8 − UPDRS at visit 1) > 1, *n* = 226)” and has now been corrected in the HTML and PDF versions of the article.

