# Supplementary material for: A blood-based DNA damage signature in patients with Parkinson’s disease is associated with disease progression
Source: Nat Aging. 2025 Sep 5;5(9):1844–61. doi: 10.1038/s43587-025-00926-x (PMC12443628; doi:10.1038/s43587-025-00926-x)
Supplement: Supplementary file 1 — Reporting Summary [file 43587_2025_926_MOESM1_ESM.pdf]

nature portfolio

Corresponding author(s): Pier G. Mastroberardino

Last updated by author(s): 2025/05/08

Reporting Summary

Nature Portfolio wishes to improve the reproducibility of the work that we publish. This form provides structure for consistency and transparency in reporting. For further information on Nature Portfolio policies, see our Editorial Policies and the Editorial Policy Checklist.

Statistics

For all statistical analyses, confirm that the following items are present in the figure legend, table legend, main text, or Methods section.

n/a	Confirmed	
☐	☒	The exact sample size (*n*) for each experimental group/condition, given as a discrete number and unit of measurement
☐	☒	A statement on whether measurements were taken from distinct samples or whether the same sample was measured repeatedly
☐	☒	The statistical test(s) used AND whether they are one- or two-sided<br>*Only common tests should be described solely by name; describe more complex techniques in the Methods section.*
☐	☒	A description of all covariates tested
☐	☒	A description of any assumptions or corrections, such as tests of normality and adjustment for multiple comparisons
☐	☒	A full description of the statistical parameters including central tendency (e.g. means) or other basic estimates (e.g. regression coefficient) AND variation (e.g. standard deviation) or associated estimates of uncertainty (e.g. confidence intervals)
☐	☒	For null hypothesis testing, the test statistic (e.g. *F*, *t*, *r*) with confidence intervals, effect sizes, degrees of freedom and *P* value noted<br>*Give P values as exact values whenever suitable.*
☒	☐	For Bayesian analysis, information on the choice of priors and Markov chain Monte Carlo settings
☒	☐	For hierarchical and complex designs, identification of the appropriate level for tests and full reporting of outcomes
☐	☒	Estimates of effect sizes (e.g. Cohen's *d*, Pearson's *r*), indicating how they were calculated

Our web collection on statistics for biologists contains articles on many of the points above.

Software and code

Policy information about availability of computer code

Data collection	No software was used to collect data for this study.
Data analysis	All codes used for the study are available at https://github.com/DNAdamageinPD/Natureageing and at https://codeocean.com/capsule/1403558/tree.<br><br>The downloaded Salmon files were imported into RStudio using the tximport package (version 1.26.1). Statistical power analysis was performed using the pwr package. Only transcripts with at least 10 reads across all samples were retained for analysis. Raw read counts were supplied to DESeq2 (version 1.40.2), which was used to perform differential expression analysis across Parkinson's disease (PD) groups and controls.<br><br>Quality Control<br>The quality of the data was assessed by examining the distribution of gene counts, library sizes, clustering in principal component analysis (PCA), and the ability to identify expected differentially expressed genes (DEGs) between males and females. To reduce noise without arbitrary filtering of low-count genes, the apeglm shrinkage estimator (version 1.20.0) was applied (as described in the Methods section). To account for medication effects, medication variables were included as covariates in the design matrix in DESeq2. The daily levodopa equivalent dose (LED) was included as an independent variable to adjust for its potential influence on observed gene set enrichment analysis (GSEA) pathways. Medication type was treated as a categorical variable. Patients were stratified into seven classes based on their medication regimen: (1) only levodopa/carbidopa, (2) only dopamine agonists, (3) levodopa/carbidopa and dopamine agonists, (4) levodopa/carbidopa and other medications, (5) levodopa/carbidopa, dopamine agonists, and other medications, (6) other medications, and (7) no medications. When the medication field was left blank in the PPMI records, patients were classified as not taking any medication.

Normalization and PCA
Log-transformation (rlog) was applied to the normalized counts to improve clustering distances for PCA. To identify low-quality samples, we performed a visual inspection of the distribution of the reads, focusing on two time points: baseline (first visit) and visit 8 (36 months after the first visit).

Differential Expression Analysis
Differentially expressed genes (DEGs) were estimated using log2 fold change, the Wald test, and false discovery rate (FDR) p-value correction, as implemented in DESeq2. A gene was considered differentially expressed if its FDR was below 0.05. We selected subjects with a diagnosis of Parkinson's disease (PD) and control subjects with and without mutations in LRRK2 G2019S (LRRK2 patients) or mutations in the GBA gene, including GBAN370S, GBAT408M, GBAE365K, GBAIVS2, GBA84GG, or GBAL444P (GBA patients). Demographic data are provided in Table S1.

Pathway Enrichment Analysis
Pathway enrichment analysis was performed using two approaches: overrepresentation analysis (ORA) and gene set enrichment analysis (GSEA) v.4.2.2. ORA was conducted via the EnrichR web tool (version 3.2) using Gene Ontology (GO) 2018 biological processes for the pre-filtered list of DEGs. Fisher's exact test was performed to assess the likelihood of obtaining at least the observed number of genes overlapping with each identified pathway. GSEA was conducted on an unfiltered, ranked list of genes. Genes in each PD group compared to controls were ranked by the level of differential expression using a signal-to-noise metric. The weighted enrichment statistic was calculated according to the formula:

res1$stat =log10(res1$pvalue)/sign(res1$log2FoldChange)

Statistical significance of the pathway enrichment score was determined using permutation testing over size-matched random gene sets. Multiple testing was controlled for using the family-wise error rate (FWER) threshold of 5%, which is more conservative than FDR.

Gene Set Databases
GSEA (version 4.2.2) was used to access the Kyoto Encyclopedia of Genes and Genomes (KEGG), Reactome pathway databases, the Hallmark Gene Set Collection, and WikiPathways (GSEA MSigDB collections) and WikiPathways (http://www.gsea-msigdb.org/gsea/msigdb/collections.jsp).
Pathway information was obtained from the KEGG database available through the Molecular Signatures Database (MSigDB; http://www.broadinstitute.org/gsea/msigdb/index.jsp) and the Hallmark Gene Set Collection (http://www.gsea-msigdb.org/gsea/msigdb/collections.jsp).

Gene Length Analysis
Gene length data (exons and introns) were retrieved using BiomaRt (version 3.17), with gene length defined as the difference between gene end and gene start. The distribution of log-transformed gene lengths for upregulated and downregulated DEGs was tested for normality using the Shapiro–Wilk test. Since all distributions deviated from normality, a Mann–Whitney Wilcoxon test was used to evaluate differences between upregulated and downregulated gene lengths in each comparison. Additionally, a kernel density plot of gene length distribution was drawn for each DEG list using the density function in R.

Chromosomal Fragile Sites Analysis
The list of chromosomal fragile sites (CFS) was downloaded from humCFS, a database of human chromosomal fragile sites: https://webs.iiitd.edu.in/raghava/humcfs/. A two-proportion z-test was performed to test whether the proportions of upregulated versus downregulated genes in CFS were different, with a significance threshold of 0.05.

FUMA Analysis
FUMA (https://fuma.ctglab.nl/) v1.5.4 was used to identify significantly upregulated or downregulated gene sets across human tissue types. The GENE2FUNC tool of FUMA was run using GTEx v8 tissue types and general tissue categories. Tissue specificity was tested using hypergeometric tests, and the direction of expression was considered with a p-value threshold of 0.1. FUMA reports gene sets with an adjusted p-value ≤ 0.05 and the number of overlapping genes by default. Data are visualized in a histogram representing the −log10(p-value), with significant results shown in red and non-significant results in blue.

For manuscripts utilizing custom algorithms or software that are central to the research but not yet described in published literature, software must be made available to editors and reviewers. We strongly encourage code deposition in a community repository (e.g. GitHub). See the Nature Portfolio guidelines for submitting code & software for further information.

Data

Policy information about availability of data

All manuscripts must include a data availability statement. This statement should provide the following information, where applicable:

- Accession codes, unique identifiers, or web links for publicly available datasets
- A description of any restrictions on data availability
- For clinical datasets or third party data, please ensure that the statement adheres to our policy

Raw data for blood transcriptome analysis and metadata used in this study are available for downloading from PPMI (Parkinson progressive markers initiative cohort). PPMI data were initially downloaded at https://ida.loni.usc.edu/pages/access/geneticData.jsp#441 on 11th May 2023 after completing a data user agreement.
Salmon files are available at the LONI IDA website (https://ida.loni.usc.edu/ pages/access/geneticData.jsp#441) under formal request with a data-use agreement.
Patient information (clinical scales assessment, DaTscan and MRI imaging, genome sequencing data, patient history, medications) are also available on the LONI IDA website.

Research involving human participants, their data, or biological material

Policy information about studies with human participants or human data. See also policy information about sex, gender (identity/presentation), and sexual orientation and race, ethnicity and racism.

Reporting on sex and gender	In comparative analysis (for brain and blood samples) both males and females were included in the analysis. Demographic tables in the supplementary section provide detailed information of the disease evolution, age and gender used in the analysis.
Reporting on race, ethnicity, or other socially relevant groupings	No information related to race, ethnicity or other socially relevant groupings has been used for this study.
Population characteristics	For both brain and blood specimens, full details in the population characteristics (age, gender, primary diagnosis, medication status) are included in figure 1A and in the supplementary tables (Supplementary figures 1A, 9A).
Recruitment	We did not perform subject recruitment.
Ethics oversight	Brain samples have been provided by the Queen Square Brain Bank for Neurological Disorders and regulated by a Material Transfer Agreement (MTA), which ensured that samples were obtained and used in accordance with legal and ethical requirements.<br>The publicly available transcriptome data from the PPMI cohort has been used according to the repository's guidelines and policies.<br>PBMCs from idiopathic PD patients were obtained from the Profiling Parkinson's Disease study. The study was approved by the medical ethics committee of the Leiden University Medical Center, and written informed consent was obtained from all PD patients.

Note that full information on the approval of the study protocol must also be provided in the manuscript.

Field-specific reporting

Please select the one below that is the best fit for your research. If you are not sure, read the appropriate sections before making your selection.

☒ Life sciences ☐ Behavioural & social sciences ☐ Ecological, evolutionary & environmental sciences

For a reference copy of the document with all sections, see nature.com/documents/nr-reporting-summary-flat.pdf

Life sciences study design

All studies must disclose on these points even when the disclosure is negative.

Sample size	Taking advantage of the PPMI resource (https://www.ppmi-info.org/), we evaluated longitudinal expression data in blood samples from 484 PD patients and 187 controls examined at the intake visit (visit 1), and in 268 and 157 of these patients and healthy control subjects (HSC) that were examined in a follow up visit after 36 months (visit 8) (fig.S1A, detailed information in Table S1 and Table S2; see also supplementary excel file “Index supplementary tables” for guidance on tables’ content).
Data exclusions	5 prodromal cases were excluded from the study because no information was available on whether they developed PD in the 2 years after visit 8. This is indicated in the main text.
Replication	The data were replicated on a two independent datasets (GSE99039 and GSE68719 datasets) available at the https://www.ncbi.nlm.nih.gov/geo website.
Randomization	In our study we focused on the analysis of Parkinson's disease progression, randomization was not employed as it is not applicable to the nature of our research. This investigation was designed to comprehensively assess the natural course and evolution of Parkinson's disease over time, without any form of intervention or experimental manipulation.
Blinding	Cell biology experiments in PBMC and immunofluorescence studies in human brains were performed in a blinded fashion. This is stated in the main text.

Reporting for specific materials, systems and methods

We require information from authors about some types of materials, experimental systems and methods used in many studies. Here, indicate whether each material, system or method listed is relevant to your study. If you are not sure if a list item applies to your research, read the appropriate section before selecting a response.

April 2023

Materials & experimental systems

n/a	Involved in the study
☐	☒ Antibodies
☒	☐ Eukaryotic cell lines
☒	☐ Palaeontology and archaeology
☒	☐ Animals and other organisms
☒	☐ Clinical data
☒	☐ Dual use research of concern
☒	☐ Plants

Methods

n/a	Involved in the study
☒	☐ ChIP-seq
☒	☐ Flow cytometry
☒	☐ MRI-based neuroimaging

Antibodies

Antibodies used

Anti -tyrosine Hydroxylase (TH – 1:500, mouse monoclonal, clone LNC1 MAB 318, Merk Millipore); anti -gamma H2AX (1:500 – rabbit polyclonal ab11174, Abcam); anti -gamma H2AX (1:1000 – mouse monoclonal, clone JBW301, 05-636, Merck Millipore); Donkey-anti-Mouse Alexa 488 secondary antibody (1:800; A-21202 Thermo Fisher); Donkey-anti-Rabbit Alexa 647 secondary antibody (1:800; A-31573 Thermo Fisher)

Validation

MAB318: https://www.merckmillipore.com/IT/it/product/Anti-Tyrosine-Hydroxylase-Antibody-clone-LNC1,MM_NF-MAB318?ReferrerURL=https%3A%2F%2Fwww.google.com%2F

ab11174: https://www.abcam.com/products/primary-antibodies/gamma-h2ax-phospho-s139-antibody-ab11174.html

ab05-636: https://www.sigmaaldrich.com/IT/it/product/mm/05636?srsltid=AfmBOoq6VWo1RHB1tfqSwrb8EFFj-Y2Wq0MIXV0e_ukb1TJiqhDfgNx1
